# Evaluating Transferability of ComBat Harmonization of Diffusion Tensor Magnetic Resonance Imaging Data

**DOI:** 10.1007/s10439-025-03886-w

**Published:** 2025-11-07

**Authors:** Bradley Fitzgerald, Thomas M. Talavage

**Affiliations:** 1 School of Engineering, Samarkand International University of Technology, Samarkand, Uzbekistan; 2 Elmore Family School of Electrical and Computer Engineering, Purdue University, West Lafayette, IN USA; 3 Department of Biomedical Engineering, University of Cincinnati, Cincinnati, OH USA

**Keywords:** Data harmonization, ComBat, Neuroimaging, DT-MRI

## Abstract

**Purpose:**

Traditional use of the ComBat data harmonization method (a popular means of harmonizing multisite MRI data) is limited by the requirement that the harmonization must be recomputed with any addition of new subjects to the data pool. The goal of this study was to assess whether a transferable ComBat (T-ComBat) algorithm could be applied such that harmonization parameters computed using ComBat on a fixed set of diffusion tensor (DT) MRI training subject data could be reapplied to harmonize new, previously unseen subject data without alteration of the harmonized training data. Emphasis was given to evaluating the necessary size of such a training data pool.

**Methods:**

Fractional anisotropy (FA) and mean diffusivity (MD) maps for 314 adolescents were harmonized across two MRI scanning sites using the T-ComBat method applied to new subject data for a variety of training group sizes. For each training group size, FA and MD maps were assessed for differences across sites after T-ComBat on voxel-wise and region-of-interest-wise (ROI) levels. Voxels and ROIs were tested for significant differences across sites.

**Results:**

T-ComBat yielded improved harmonization across sites but did not reach the performance of full ComBat. A limited number of new subjects (approximately 25% for FA, 10% for MD) could be harmonized via T-ComBat and still yield a sufficiently harmonized total dataset.

**Conclusion:**

T-ComBat may be applied to harmonize DT-MRI data from a limited number of new subjects from previously seen scanners without necessitating the reharmonization of previously analyzed data.

**Supplementary Information:**

The online version contains supplementary material available at 10.1007/s10439-025-03886-w.

## Introduction

Diffusion-weighted magnetic resonance imaging (DW-MRI) is a powerful, noninvasive medical imaging modality used to study the diffusion of fluids in the human brain. Specifically, diffusion tensor imaging (DTI) via MRI is used to compute voxel-wise diffusion tensors that describe the orientation, magnitude, and degree of anisotropy of water molecule diffusion throughout the brain [1, 2]. DTI has been proven useful in the study of a variety of brain conditions, including neurodegenerative disorders [3–5], stroke [6, 7], traumatic brain injury [8–10], and others [11, 12]. A shortcoming of DW-MRI is that its collection is time-consuming and expensive, which limits the number of participants that can be feasibly imaged on a single MRI scanner for any given study. In response to the need for greater study power through larger sample sizes, multisite MRI studies and publicly available datasets are becoming increasingly prevalent. However, pooling of MRI data from multiple locations and scanners is limited by noise introduced by individual scanners. Specifically, metrics derived from DTI via MRI have been shown to vary depending on a range of scan sequence parameters such as spatial and angular resolution [13, 14], number of diffusion-weighting directions [15], and the chosen b-values [16]. To ensure that accurate results may be derived from multisite DTI studies, it is critical that methods of multisite DTI data harmonization are developed and thoroughly evaluated.

A number of different methods have been proposed to reduce the impact of scanner-induced noise in DTI data [17, 18]. Some methods aim to reduce sources of scanner variation in the image domain by using same-vendor scanners with standardized pulse sequence parameters [19–21], by standardizing analysis protocols [22, 23], or through emerging machine-learning methods for harmonization of raw image data [24]. However, standardized acquisition and analysis can still result in significant differences in diffusion-related metrics between sites [25]. In response, more methods have been explored to harmonize diffusion metrics in the feature or metric domain [18, 26]. These methods include surrogate variable analysis [18, 27, 28], site-wise harmonization of location-scale parameters [27], linear regression-based models [29], the use of a traveling phantom [30], or generative adversarial networks [31].

Recently, a data harmonization method known as ComBat, which was originally developed for the removal of batch effects from multi-batch gene expression data [32], was demonstrated to be effective at removing scanner-specific variability in fractional anisotropy (FA), mean diffusivity (MD), and cortical thickness measurements derived from multisite MRI neuroimaging [27, 33]. ComBat estimates additive and multiplicative site effect parameters using an empirical Bayes (EB) framework while relying on an assumption of common parametric prior distributions and is a robust method for multisite harmonization of data with modest sample size [27]. Since its introduction into medical imaging by Fortin et al. [27], there has been a sharp increase in the number of studies using ComBat to harmonize multisite medical imaging data [34]. ComBat has been applied to harmonize multisite data in MRI studies of brain functional connectivity [35–37], volumetrics [38–41], cortical thickness [42–46], spectroscopy [47], and diffusivity measures (such as FA and MD) [48–50]. ComBat has also been adopted in various multisite radiomics studies utilizing positron emission tomography (PET) and computed tomography (CT) [51–55]. Further, several altered versions of the ComBat algorithm have been proposed for various specific applications [43, 56].

Despite its popularity, current use of ComBat suffers from a limitation in that it requires re-computation of the harmonizing transformation after the addition of any new subjects to the data pool, which is undesirable for larger, long-term multisite collaborations in which researchers may wish to run multiple analyses over the course of years of data collection. This raises the question of whether ComBat can be used to develop a harmonizing transformation that is transferable to new subjects (scanned on a previously seen scanner), such that new subject data can be harmonized without changing data that was previously harmonized. Beer et al. have proposed an altered ComBat algorithm for applications to longitudinal studies [43], allowing for statistical parameter estimates that vary with time, but this method is still designed for application at the end of all multisite data collection. This concept of a transferable ComBat harmonization has received brief attention from Da-ano et al. [57], who tested the transferability of the harmonization of multicenter radiomics data and concluded that the method was capable of using a predetermined harmonizing transform to harmonize radiomic features from new subjects from a known scan center with slightly decreased performance. However, it remains unclear under what conditions the transfer of a predetermined ComBat harmonization to new data is acceptable, particularly with respect to the number of data points included in the original dataset and the number of data points to be added.

Therefore, the current study aimed to evaluate the transferability of ComBat harmonization—computed based on an assumed pre-existing “training” dataset—applied to harmonize new data from known collection sites without impacting the harmonization of the pre-existing harmonized training data. We refer to this method as transferable ComBat (T-ComBat). T-ComBat was applied to harmonize DTI measures of FA and MD across sites using an adolescent MRI dataset collected across two different scan sites. Repeated subsampling of the data pool and application of T-ComBat were conducted, with the harmonizing performance assessed as a function of the number of subjects included in the original training dataset and the number of new subjects added to the data pool. The results of these analyses provide guidelines for the conditions under which T-ComBat may be reasonably applied to add new subject DTI data to a site-harmonized pool.

## Materials and Methods

### Participants and Data Acquisition

Data utilized in this study included T1-weighted, T2-weighted, and DW-MR images collected and shared by the National Consortium on Alcohol and Neurodevelopment in Adolescence—Adulthood (NCANDA-A; https://ncanda.org/). NCANDA-A data were selected for this study due to its large pool of participants scanned at multiple sites. The procedures used in this study were in accordance with the Declaration of Helsinki. All participant data were collected under IRB approval at the collection sites; adult participants provided informed consent, and minors provided written assent and the consent of a legal guardian [58]. A detailed description of the dataset, scan protocols, and imaging coils can be found in previous publications [58, 59]. The full dataset collected by NCANDA-A included participants recruited from five different sites in the United States, with each site utilizing either a General Electric (GE) 3 T Discovery MR750 or a Siemens 3 T TIM TRIO MRI scanner. Data from just two of these sites—University of California San Diego (UCSD) and Oregon Health and Sciences University (OHSU)—were selected to be used for the current study. UCSD was selected as the GE scanner site, as it had the largest number of participants on this platform, and OHSU was selected as the Siemens scanner site, as it had the largest number of participants on that platform. All imaging data provided by NCANDA-A underwent quality control checks including automatically generated scores and radiologist reports [58].

MRI data for a total of 314 adolescent participants (180 UCSD, 134 OHSU), 12–21 years of age, passed in-house image quality checks (described in a subsequent section, additional to those already performed by NCANDA-A) and were included in the current study. All included participants met NCANDA-A’s criteria for no/low alcohol consumption [58] (any participant that exceeded such consumption criteria was excluded). Table 1 displays participant demographics including age, sex, and self-declared ethnicity. To ensure that participants from the two sites were demographically similar, the following three statistical tests were performed: (1) a two-sample t-test comparing age across sites, (2) a binomial test comparing the proportion of male/female subjects across sites, and (3) a chi-squared test of independence comparing proportions of declared ethnicities across sites. The age and sex-related tests did not yield statistical significance (*p* > 0.05 for both), while the chi-squared test relating to ethnicity did signify a statistically significant difference across sites (*p* = 0.008). Despite this difference in ethnicity distribution across sites, later analysis demonstrated that ethnicity did not significantly impact the FA and MD values assessed, so the two sites were deemed to be demographically similar for the purposes of this study.

**Table 1:** Demographics of included participants (n = 314)

	UCSD participants (n = 180)	OHSU participants (n = 134)
Age (mean ± st. dev.)	16.0 ± 2.4 years	16.2 ± 2.6 years
Sex	93 male, 87 female	67 male, 67 female
Self-declared ethnicity		
Native American/American Indian	1	1
Asian	18	11
Pacific Islander	0	3
African American/Black	11	1
Caucasian/White	128	111
Other	22	7

### Image Preprocessing

MRI processing was conducted using software from the FMRIB Software Library (FSL) [60] and Advanced Normalization Tools (ANTs) [61]. T1-weighted images underwent denoising (filters described by [62–64]) and bias correction (FSL *fsl_anat*). Brain voxels were isolated within the images via three methods: (1) a marker-based watershed scalp extraction program [65] applied to the T1 image; (2) brain extraction using FSL’s *BET* applied to the T1-weighted image; and (3) brain extraction using FSL’s BET applied to the T2-weighted image, which was then registered to the T1-weighted image using FSL’s FLIRT [66]*.* The final brain mask was defined by including voxels isolated by the majority of the three listed methods. The extracted T1-weighted brain mask was aligned via linear registration (FSL *flirt* with only translational and rotational adjustments) to the space of the *b* = 0 DW image (for use during DW data processing and to establish a transformation for alignment between the T1-weighted images and the DW images). The extracted T1-weighted brain image was also nonlinearly registered to the ICBM 152 1 mm isometric brain atlas [67] (ANTs *antsRegistrationSyNQuick.sh*), establishing a transformation for alignment between the T1-weighted images and the standard template space. These two transformations were saved and used in conjunction to register each subject’s FA and MD maps to the standard atlas space to facilitate voxel-wise and region of interest (ROI)-wise data comparisons across subjects.

DW-MRI images underwent correction of susceptibility induced distortions (FSL *topup* [68]) followed by correction for eddy currents and movement (FSL *eddy* [69]). For quality control, subjects were not included if their motion statistics (computed by FSL *eddy*) reflected average between-volume motion greater than 3 standard deviations from the mean average motion of all subjects or if their maximum root mean square motion between volumes exceeded 5 mm [9]. A total of 180 participants from UCSD and 134 participants from OHSU with no or low alcohol use history, whose data passed quality control, were included in the subsequent analyses. The corrected DW images were used to fit diffusion tensors and estimate voxel-wise fractional anisotropy (FA) and mean diffusivity (MD) maps (FSL *dtifit*). The generated FA and MD maps were nonlinearly registered to the ICBM 152 standard space using the saved transformations. This registration aligned all FA and MD within a common image space to facilitate voxel-wise and ROI-wise data comparisons across subjects.

### Determination of Covariates of Interest

**Fig. 1:**
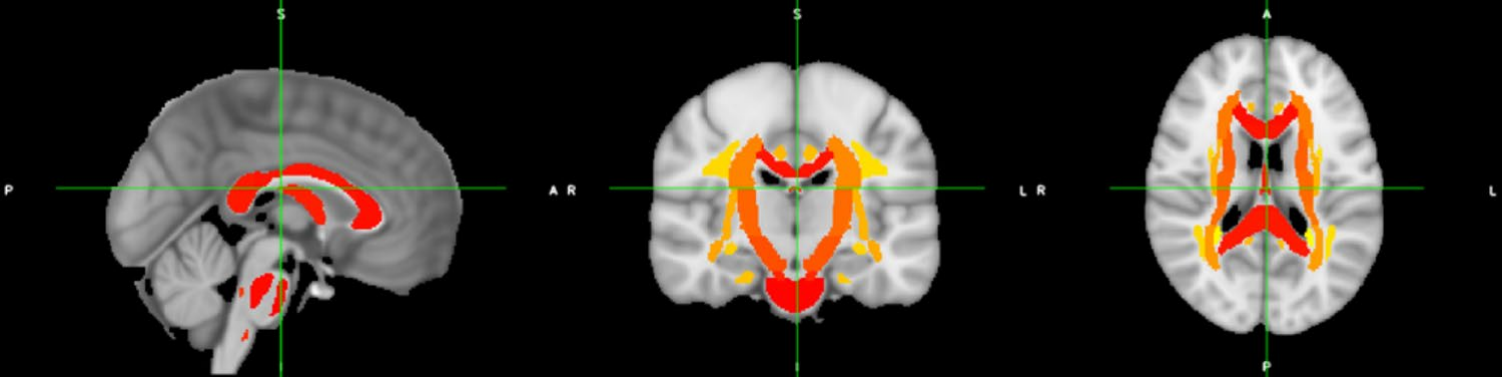
JHU white matter atlas [70] (red-yellow) overlaid on ICBM152 standard atlas [67]. The JHU atlas contains 48 ROIs and was used to mask WM voxels across all standardized FA maps

To identify which biological factors should be considered when conducting ComBat harmonization, the Johns Hopkins University (JHU) white matter (WM) atlas [70] was used as a mask to isolate select WM voxels across all subjects’ standardized FA and MD maps (Fig. 1). Though the JHU WM atlas contains 48 labeled ROIs, all voxels included within all 48 ROIs were included to form the WM mask. For each subject, FA was averaged over this WM mask to produce average WM FA values. Multiple linear regression was conducted to assess the statistical dependence of WM average FA values on independent variables including site, age, sex, and ethnicity. The same process was conducted for MD data. Only site, age, and sex were found to have a significant relationship with FA, while only site and age were found to have a significant relationship with MD. A partial F test was conducted (for both FA and MD) comparing the regression model with and without ethnicity included. The test produced *p* > 0.05 for both FA and MD, indicating that the ethnicity variable was not significant; therefore, it was excluded from subsequent analyses.

### Overview of T-ComBat Harmonization

Before describing the specific analyses conducted, here we first give a general description of data harmonization using ComBat. ComBat is formulated for harmonization of datasets collected from multiple sources. In the case of this study, we assume that data, in the form of FA maps, originates from one of multiple different scanners. We let ${y}_{ijv}$ represent the observed FA measure for voxel $v$ from subject $j$ scanned at site $i$. ComBat assumes the following model for observed data:

1 $$y_{ijv} = \alpha_{v} + {\boldsymbol{X}}_{ij} {\boldsymbol{\beta}}_{v} + \gamma_{iv} + \delta_{iv} \varepsilon_{ijv}$$

where ${\alpha }_{v}$ is the overall FA value for voxel $v$, ${\boldsymbol{X}}_{ij}$ is a design vector for relevant covariates (such as age or sex) corresponding to subject $i$ and site $j$, ${\boldsymbol{\beta}}_{v}$ is a voxel-specific vector of regression coefficients associated with ${\boldsymbol{X}}_{ij}$, $\gamma_{iv}$ is the additive site effect for site $i$ and voxel $v$, $\delta_{iv}$ is the multiplicative site effect for site $i$ and voxel $v$, and $\varepsilon_{ijv}$ represents an error term which follows a zero-mean normal distribution with variance $\sigma_{v}^{2}$ [27]. First, estimates of $\hat{\alpha }_{v}$, $\hat{\sigma }_{v}$, and $\hat{\boldsymbol{\beta }}_{v}$ are computed according to ordinary least-squares regression. “Standardized” data are computed based on these estimates as follows:

2 $$z_{ijv} = \frac{{y_{ijv} - \hat{\alpha }_{v} - {\boldsymbol{X}}_{ij} \hat{\boldsymbol{\beta }}_{v} { }}}{{\hat{\sigma }_{v} }}$$

after which the standardized data $z_{ijv}$ are assumed to be normally distributed according to $z_{ijv} \sim N\left( {\gamma_{iv} , \delta_{iv}^{2} } \right)$. Combat assumes that the site effect parameters $\gamma_{iv}$ and $\delta_{iv}$ have parametric prior distributions:

3 $$\gamma_{iv} \sim N\left( {\gamma_{i} ,\tau_{i}^{2} } \right),\quad \delta_{iv}^{2} \sim {\text{ Inverse}}\;{\mathrm{Gamma}}\left( {\lambda_{i} , \theta_{i} } \right)$$

where estimates of the hyperparameters ${\gamma }_{i}$, ${\tau }_{i}^{2}$, ${\lambda }_{i}$, and ${\theta }_{i}$ are computed via the method of moments. Finally, voxel-specific, empirical Bayes estimates of the site effects are computed as ${\gamma }_{iv}^{*}$ and ${\delta }_{iv}^{*}$ using conditional posterior means as described by [32]. Thus, the full ComBat harmonization transformation is given as:

4 $$y_{ijv}^{{{\mathrm{ComBat}}}} = \frac{{\hat{\sigma }_{iv} { }}}{{\hat{\delta }^{*}_{iv} }}\left( {z_{ijv} - \gamma_{iv}^{*} } \right) + \hat{\alpha }_{v} + {\boldsymbol{X}}_{ij} \hat{\boldsymbol{\beta }}_{v}$$

where ${y}_{ijv}^{\mathrm{ComBat}}$ represents the harmonized FA measure.

It is of interest to evaluate whether the parameter estimates computed using the ComBat harmonization method can be applied to harmonize data for new, previously unseen subjects (scanned on previously seen scanners). To this end, in this study, we assess a transferable ComBat methodology (hereafter referred to as T-ComBat) in which ComBat is first applied to harmonize data for a fixed subset of observed subjects (referred to as the training group), producing estimates of the model parameters $\hat{\alpha }_{v} , \hat{\sigma }_{iv} , \hat{\boldsymbol{\beta }}_{v} , \gamma_{iv}^{*} ,$ and $\hat{\delta }^{*}_{iv}$, which are saved. These saved parameters are then applied to harmonize data for a new subset of observed subjects (referred to as the test group) not included in the training group. The harmonizing performance of T-ComBat was evaluated using several analyses described in subsequent sections.

### Analyses of T-ComBat Harmonization Performance

Several analyses were completed with the goal of assessing the performance of T-ComBat with varying numbers of subjects included in both the training and test groups. For each analysis, multiple iterations of random subject sampling from the full 314-subject pool were conducted. For each such iteration, a chosen number, ${n}_{Train}$, of training subjects were randomly selected for each of the two sites; a chosen number, ${n}_{Test}$, of test subjects were then randomly selected from the remaining subjects for each of the two sites. Whole-brain FA or MD maps for the selected training and testing groups were vectorized and harmonized using the T-ComBat method with site, sex, and age entered as covariates. For brevity, the following analyses are described in terms of FA map harmonization, but we note that each of these analyses was conducted twice—once using FA maps and once using MD maps.

### Test Group Harmonization as Training Group Size Varies

This analysis was designed to assess the harmonizing performance of T-ComBat on a fixed-size test group (${n}_{Test}=20$ from each site) while the size of the training group was varied with ${n}_{Train}\in \{50, 60, \dots , 110\}$ from each site. For each value of ${n}_{Train}$, 1000 iterations of randomly selecting the training and test groups harmonized via T-ComBat were conducted. For each iteration, harmonization of the test group FA maps was assessed on two levels:

*Voxel-Level Assessment* For each voxel within the JHU WM atlas, harmonized test group FA values for UCSD subjects were compared against those of OHSU subjects using a two-sample* t*-test with significance when *p* < 0.05 (as used previously by Fortin et al. [27]). Note that a multiple comparison correction was not warranted to correct for multiple voxels being assessed, as the purpose of this assessment was specifically to evaluate *how many* voxels exhibited statistically significant differences across site as opposed to assessing which particular voxels remained unharmonized. The percentage of voxels displaying a significant difference across sites (relative to the total number of voxels within the JHU WM atlas) was recorded. The collection of 1000 observations of this percentage formed the voxel-level harmonizing performance distribution of T-ComBat for the given ${n}_{Train}$ size.

*ROI-Level Assessment* The JHU WM atlas’ 48 ROIs were used as masks to isolate the 48 ROIs within each subject’s FA and MD maps. The harmonized FA maps of each subject were averaged within each of the 48 ROIs. ROI-averaged FA values were then compared across sites in the same manner as voxel-wise FA .

In addition to its harmonizing performance, it is also of interest to observe the behavior of T-Combat with regard to maintaining statistical association between FA and MD with biological covariates such as age and sex. Rates of such association were observed during the analysis just described, with details and results presented in the Supplementary Material (SM) Section 1.

To interpret the harmonizing performance of T-ComBat, it is of interest to evaluate the FA differences across site at these two levels (voxel and ROI) for two “baseline” scenarios: (1) using the unharmonized FA maps and (2) using the FA maps after the entire UCSD and OHSU datasets have been passed through the traditional ComBat algorithm, with (2) representing the ideal performance of ComBat on the dataset. Given that the likelihood of obtaining a statistically significant *p*-value is affected by the number of subjects included in the test, and since the T-ComBat performance was assessed on groups consisting of 20 test subjects per site, a repeated procedure was applied to assess these two baseline performance levels using 20 subjects per site. To evaluate (1), 5000 iterations were performed in which, for each iteration, 20 subjects’ FA maps from each site were randomly selected and used to assess rates of voxel-wise and ROI-wise FA difference across sites in the same manner (using two-sample *t*-tests) described previously. To evaluate (2), the same procedure was applied after the entire 314-subject dataset was harmonized using ComBat.

For each level of assessment, this produced a distribution of 5000 full ComBat performance samples (each sample representing a percentage of voxels or ROIs with significant difference across sites) that could be directly compared against the 1000-sample distribution of T-ComBat performance measures at each training group size. These performance distributions were compared using two metrics. First, for each $n{}_{Train}$ size, the proportion (out of all 1000 iterations) was computed of T-ComBat voxel-wise (or ROI-wise) performance measures less than the 95^th^ percentile of the same performance measure of the full ComBat trials. This first metric is hereafter referred to as the *proportion within full ComBat* performance ($PwFC$). Second, the overlap between the voxel-wise (or ROI-wise) T-ComBat performance distribution and the voxel-wise full ComBat performance distribution was assessed using the overlapping index $\eta$ (with $\eta$ computed as defined by Pastore et al. [71] following estimation of probability density functions using MATLAB *gkdeb* [72]) between the two distributions.

### Combined Training and Test Group Harmonization as Test Group Size Varies

The analyses described above provide a useful perspective on the harmonizing performance of T-ComBat as measured on a fixed-size group of test subjects with varying training group sizes. However, T-ComBat would likely be applied in scenarios where the “new” test subjects were added to the existing training cohort and the entire, combined dataset was used to run certain analyses. From this perspective, it is also beneficial to assess the harmonization of the combined (training and test) dataset with varying numbers of test group sizes, as this would reveal the relative number of test subjects that could be added to an existing cohort while still expecting acceptable harmonization via T-ComBat.

To this end, an additional iterative analysis was conducted. For values of ${n}_{\mathrm{Train}}\in \{50, 60, \dots , 100\}$, it was intended to approximate the maximum number of test subjects (i.e., not initially included in the ComBat harmonization parameter computation) that could be added to the training cohort and maintain complete voxel-wise harmonization of the combined dataset. For each ${n}_{\mathrm{Train}}$ size, values of ${n}_{\mathrm{Test}}\in \left\{2, 5, 10, 15, 20, 25\right\}$ were assessed by beginning with a lower ${n}_{Test}$ and increasing until full harmonization was no longer achieved. For each value of ${n}_{\mathrm{Test}}$, 500 trials were conducted involving random selection of ${n}_{\mathrm{Train}}$ training subjects and ${n}_{\mathrm{Test}}$ test subjects, applying T-ComBat, and then testing all JHU WM voxels to assess the percentage which still demonstrated significant differences across sites using a voxel-wise two-sample *t*-test (at the *p* < 0.05 level). For every tested combination of ${n}_{\mathrm{Train}}$ and ${n}_{\mathrm{Test}}$, T-ComBat was determined to completely harmonize the voxel-wise FA or MD data if at least 95% of the iterations produced less than 5% of voxels exhibiting significant differences (after T-ComBat harmonization) across sites.

### Assessing Prevalence of Differences across Groups Sampled from Same Site

The previous analyses demonstrated that T-ComBat performance did not reach the full level of performance of full ComBat applied to the entire dataset (described in detail in the Results). Another analysis was conducted to validate this result with an additional measure of the baseline similarity of WM FA and MD values expected from subjects within the same site. For each of the two sites (separately), 5000 repeated trials were conducted in which two groups of 20 subjects were randomly selected from within the same site and voxel-wise WM FA values were tested for significant differences across the two groups (using two-sample* t-*tests, *p* < 0.05). The resulting distributions of significant difference prevalence for each site were tested for differences against the distribution resulting from full ComBat applied to the whole dataset (Wilcoxon rank sum tests, *p* < 0.05).

## Results

### Determination of Covariates of Interest

Table 2 presents statistics related to the two multiple linear regression analyses modeling FA and MD (averaged over all voxels within the JHU WM mask) against site, sex, and age. Site and age were found to have significant (*p* < 0.05) associations with both average FA and average MD. Sex was found to have a significant association with average FA but not with average MD. The partial F tests (conducted to assess whether including ethnicity as a covariate significantly added to the regression model) yielded *p* > 0.05, so ethnicity was excluded from the regression analyses reported in Table 2.

**Table 2:** Results of multiple linear regression assessing the association of site, sex, and age with fractional anisotropy and mean diffusivity (each averaged over all voxels in the JHU white matter atlas) based on all 314 subjects from the combined UCSD and OHSU datasets (before harmonization)

		Coefficients	Standard Error	t Stat	*p*-value
Fractional anisotropy (FA) regression	Intercept	0.456	0.00621	73.4	***p* < 0.001**
	Site	− 0.0201	0.00186	− 10.8	***p* < 0.001**
	Sex	0.00790	0.00184	4.30	***p* < 0.001**
	Age	0.00191	0.000368	5.184	***p* < 0.001**
Mean diffusivity (MD) regression	Intercept	0.000890	9.83E−06	90.6	***p* < 0.001**
	Site	2.38E−05	2.94E−06	8.12	***p* < 0.001**
	Sex	− 1.99E−06	2.90E−06	− 0.686	0.4593
	*Age*	− 1.84E−06	5.82E−07	− 3.166	0.**00170**

Covariates with statistically significant (*p* < 0.05) influence are bolded

### Analyses of T-ComBat Harmonization Performance

**Fig. 2:**
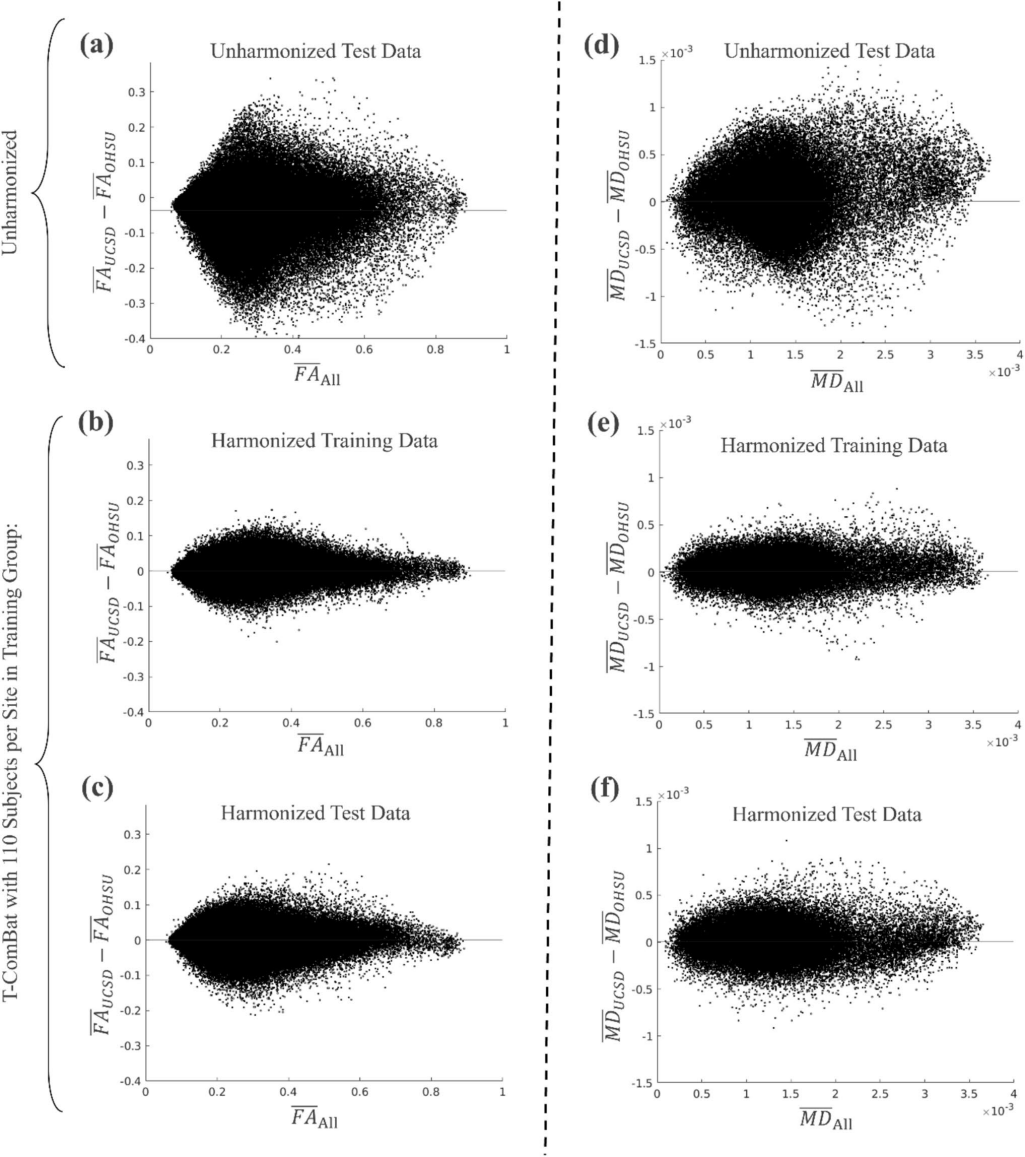
Example voxel-wise difference in FA (left) and MD (right) across sites before and after single instance of T-ComBat harmonization. Scatterplots on the left represent voxel-wise FA averaged over all subjects included in the training or test group ( ${\overline{FA} }_{All}$, horizontal axes) versus voxel-wise FA, averaged over the included UCSD subjects, minus that averaged over included OHSU subjects (${\overline{FA} }_{\mathrm{UCSD}}-{\overline{FA} }_{\mathrm{OHSU}}$, vertical axes). Horizontal black lines indicate the mean value of ${\overline{FA} }_{\mathrm{UCSD}}-{\overline{FA} }_{\mathrm{OHSU}}$ averaged over all voxels. **a** Differences in FA across sites for 20 subject per site test group before harmonization. **b** Differences in FA across sites for training group after T-ComBat applied with 110 subjects included in training group. The figure was created using a random sampling of 20 subjects per site from the harmonized training group so that the resulting scatterplot would be comparable to **c**, which was created based on the 20 test subjects per site after T-ComBat. **c** Differences in FA across sites for test group (20 subjects per site) after T-ComBat applied with 110 subjects included in training group. Panels **d**–**f** represent the same data as **a**–**c** but for MD

Figure 2 showcases an example of voxel-wise difference in FA (averaged over all subjects for a given site) across the two sites (${\overline{\mathrm{FA}} }_{\mathrm{UCSD}}-{\overline{\mathrm{FA}} }_{\mathrm{OHSU}}$) before harmonization (Fig. 2a) and after application of T-ComBat (Fig. 2b, c). Specifically, Fig. 2b and c illustrate the voxel-wise difference in FA across the two sites after a single instance of applying T-ComBat with ${n}_{\mathrm{Train}}=110$ subjects per site and ${n}_{\mathrm{Test}}=20$ subjects per site. Note that Fig. 2b was created using a sampling of 20 subjects per site from the training group after T-ComBat was performed using the full 110 subjects per site so that the scatterplot would be appropriately comparable with Fig. 2a and c. Similar results for MD are presented in Fig. 2d–f.

### Test Group Harmonization as Training Group Size Varies

**Fig. 3:**
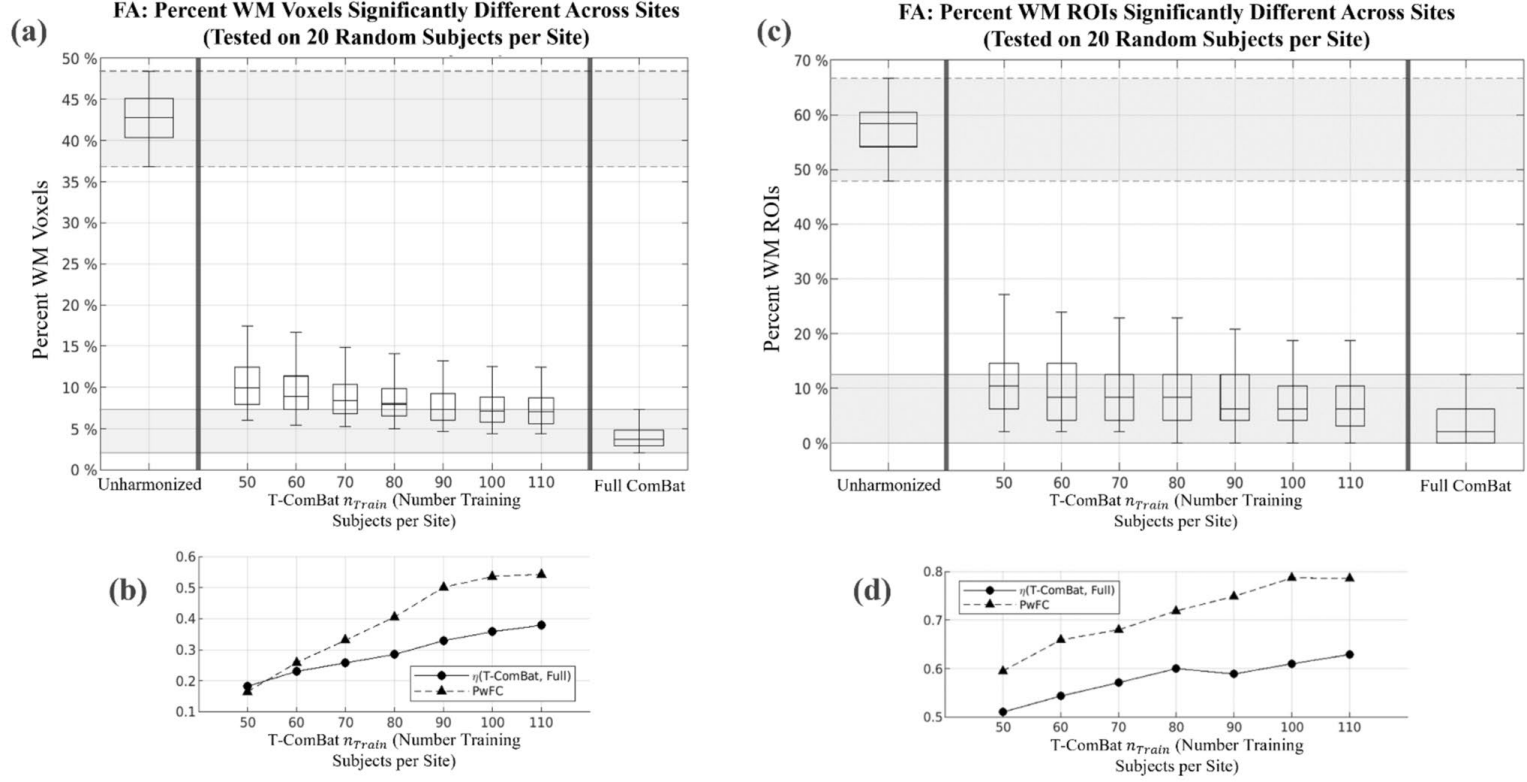
Voxel-wise and ROI-wise FA harmonization performance of T-ComBat with varying training group sizes. **a** Boxplots illustrate the results of 1000 iterations in which the percentage of JHU WM voxels with statistically significant FA difference across sites was measured on a (randomly selected) test group of 20 subjects per site. This was assessed before any harmonization (left), after T-ComBat harmonization with a given number of training subjects (middle), and after full ComBat applied to the entire 314-subject dataset (right). Lower and upper boxplot whiskers indicate 5^th^ and 95^th^ percentiles of each distribution, respectively. **b** Measures of overlap between T-ComBat performance distributions and the full ComBat performance distribution. $PwFC$ represents the proportion of T-ComBat iterations producing performance below the 95^th^ percentile of the full ComBat performance distribution, while $\eta$ represents the overlap index between the T-ComBat and full ComBat performance distributions. **c** Boxplots illustrate results similar to (**a**), except with difference across sites assessed on the ROI level (i.e., for FA averaged over each ROI from the JHU WM atlas). **d** Measures of overlap between T-ComBat and full ComBat performance distributions at the ROI level of assessment (metrics described in (**b**))

**Fig. 4:**
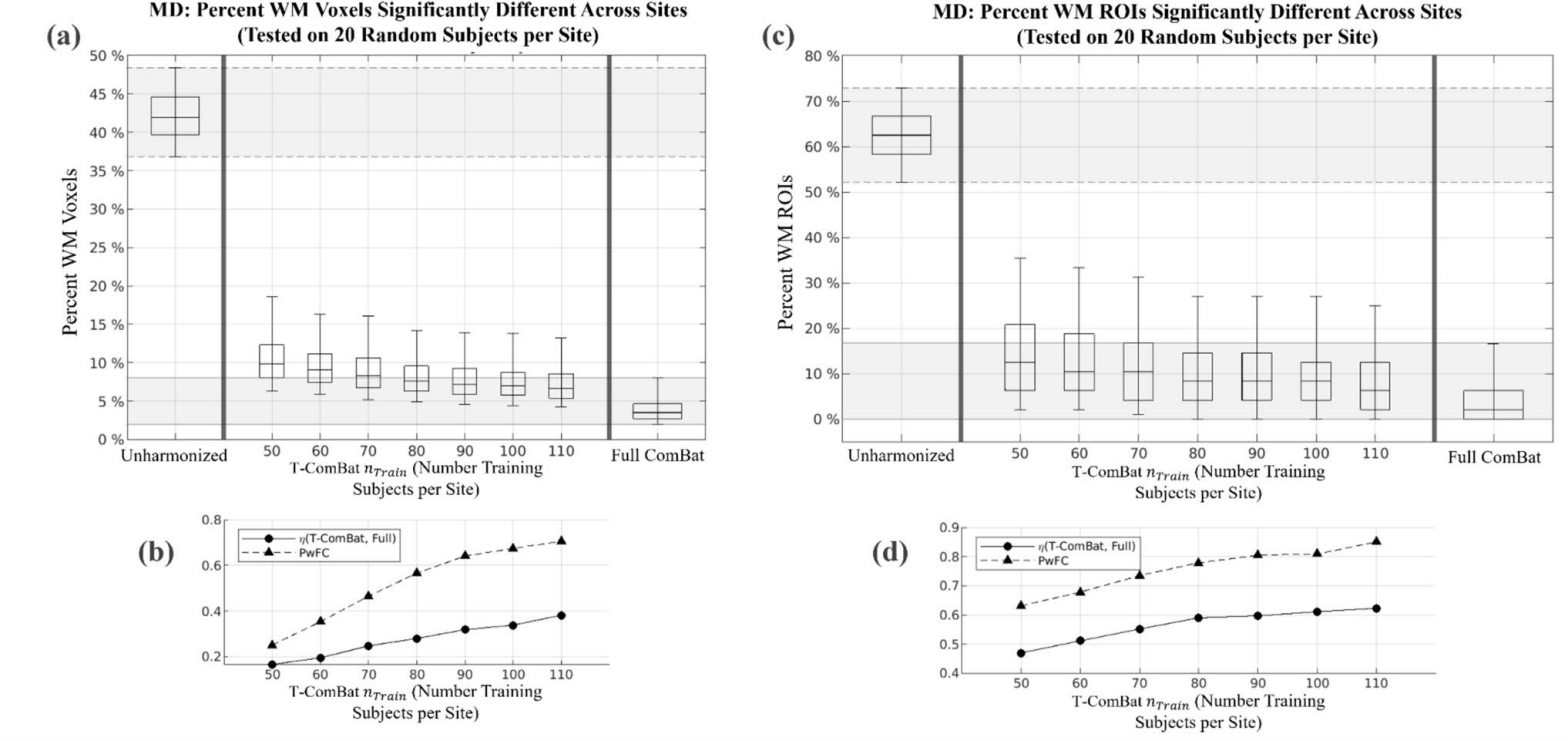
Voxel-wise and ROI-wise MD harmonization performance of T-ComBat with varying training group sizes. **a** Boxplots illustrate the results of 1000 iterations in which the percentage of JHU WM voxels with statistically significant MD difference across sites was measured on a (randomly selected) test group of 20 subjects per site. This was assessed before any harmonization (left), after T-ComBat harmonization with a given number of training subjects (middle), and after full ComBat applied to the entire 314-subject dataset (right). Lower and upper boxplot whiskers indicate 5^th^ and 95^th^ percentiles of distribution, respectively. **b** Measures of overlap between T-ComBat performance distributions and the full ComBat performance distribution. $PwFC$ represents the proportion of T-ComBat iterations producing performance below the 95^th^ percentile of the full ComBat performance distribution, while $\eta$ represents the overlap index between the T-ComBat and full ComBat performance distributions. **c** Boxplots illustrate the results similar to (a), except with difference across sites assessed on the ROI level (i.e., for MD averaged over each ROI from the JHU WM atlas). **d** Measures of overlap between T-ComBat and full ComBat performance distributions at the ROI level of assessment (metrics described in (**b**))

Figure 3 displays the performance of T-ComBat applied to harmonize FA across sites, with varying ${n}_{Train}$, based on the iterative sampling method described in the Methods. These plots demonstrate that T-ComBat achieved improved similarity between sites as compared to the unharmonized data, with improved performance as ${n}_{Train}$ increased. However, T-ComBat did not fully reach the level of performance achieved by full ComBat (i.e., when the entire dataset is used to compute the ComBat harmonization parameters). T-ComBat achieved performance that was closer to that of full ComBat when (1) it was applied to MD data compared with FA data and (2) when assessing ROI-averaged FA or MD compared with voxel-wise metrics, as indicated by higher measures of the overlap index $\eta$ and $PwFC$ metrics under these conditions. Figure 4 displays the same results for MD.

**Fig. 5:**
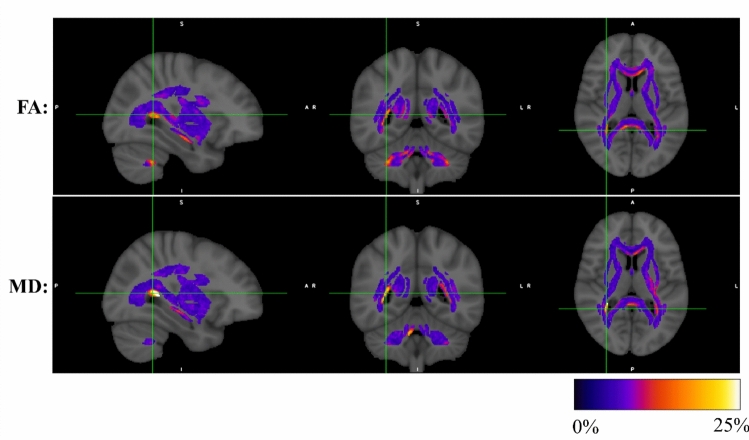
Frequency of unharmonized JHU WM voxels after T-ComBat using 110 training subjects per site. The frequencies reported represent the percentage of 1000 trials for which the voxel-wise FA (top) or MD (bottom) values of the test group (20 subjects per site) remained unharmonized (i.e., two-sample *t*-test comparing FA or MD across sites produced *p* < 0.05) after T-ComBat was applied with a training group of 110 subjects per site. Green crosshairs in each image indicate the position of slices shown. Only voxels within the JHU WM atlas were assessed and are displayed overlaid on the ICBM152 standard atlas [67]

Figure 5 displays a colormap of JHU WM voxels illustrating the frequency with which each individual voxel remained unharmonized in the test group based on the 1000 trials of T-ComBat run at the ${\mathrm{n}}_{\mathrm{Train}}=110$ training group size, for both FA and MD. Areas of the JHU atlas which bordered regions of cerebrospinal fluid (CSF), particularly toward the posterior end of the lateral ventricles, were most likely to remain unharmonized after T-ComBat.

### Combined Training and Test Group Harmonization as Test Group Size Varies

**Fig. 6:**
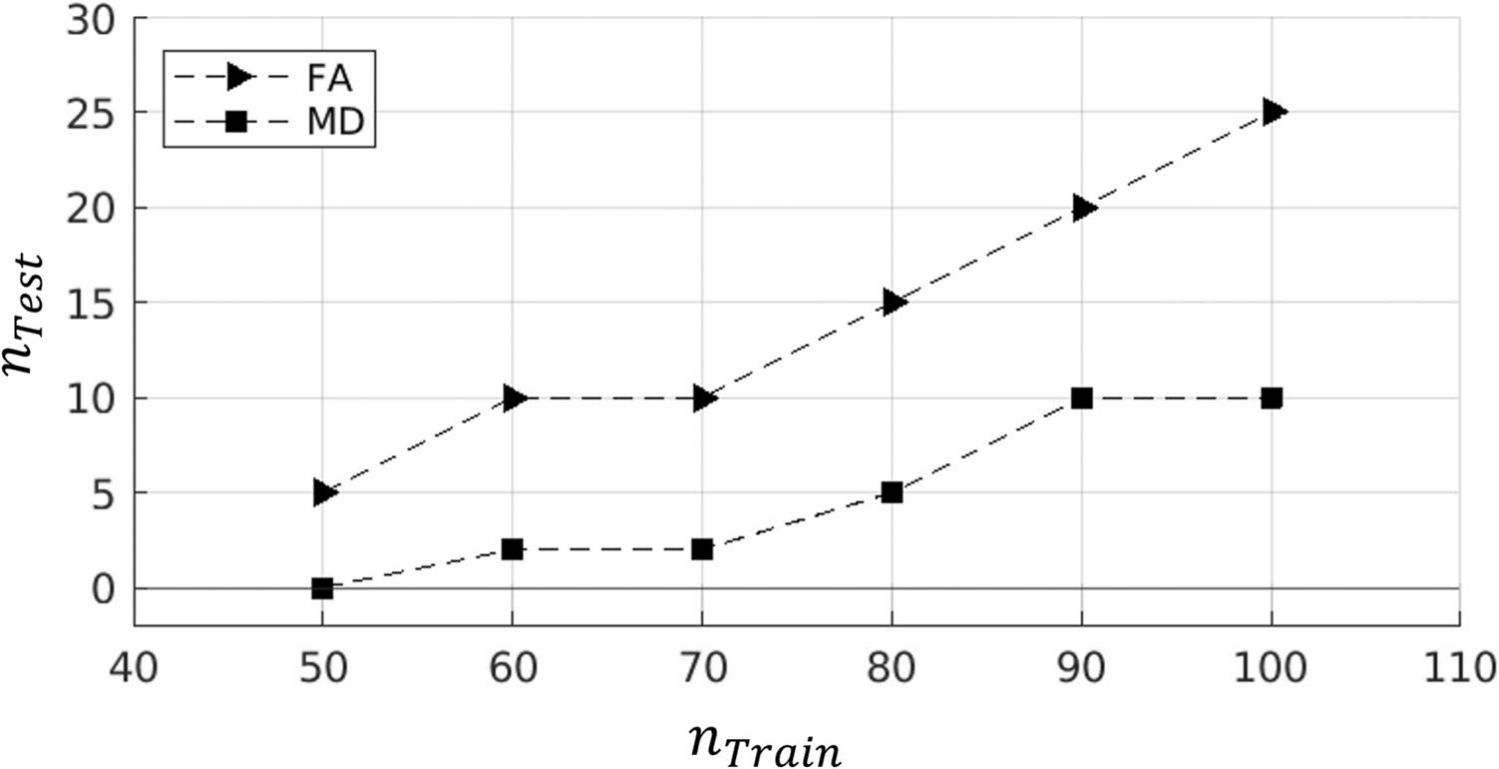
Maximum number of new “test” subjects per site (${n}_{Test}$) that can be added to the original number of “training” subjects per site (${n}_{\mathrm{Train}}$) via T-ComBat while demonstrating complete harmonization of the total combined (training + test) dataset. For each ${n}_{\mathrm{Train}}$ value shown, assessments were conducted for ${n}_{Test}\in \{2, 5, 10, 15, 20, 25\}$. Plot markers represent the maximum ${n}_{\mathrm{Test}}$ from within this set for which 95% of 500 repeated trials produced less than 5% of voxels exhibiting statistically significant difference across sites (via two-sample *t-*test, *p < *0.05) after application of T-ComBat to randomly sampled training and test subjects

Iterative analyses were conducted to evaluate the FA and MD harmonization performance of T-ComBat as assessed on a pooled cohort of both the training subjects and the test subjects, for varying sizes of ${\mathrm{n}}_{\mathrm{Train}}$ and ${\mathrm{n}}_{\mathrm{Test}}$. For each tested ${\mathrm{n}}_{\mathrm{Train}}$, the maximum number of training subjects per site (${\mathrm{n}}_{\mathrm{Test}})$ which could be added to the data pool while maintaining complete harmonization is displayed in Fig. 6. In general, for FA, T-ComBat was able to produce successful harmonization of the combined training and test groups if the test group size was about 20% or less than that of the training group. Meanwhile, when applied to MD, T-ComBat allowed for about a 10% or less addition to the original subject pool via ComBat.

### Assessing Prevalence of Differences across Groups Sampled from Same Site

**Fig. 7:**
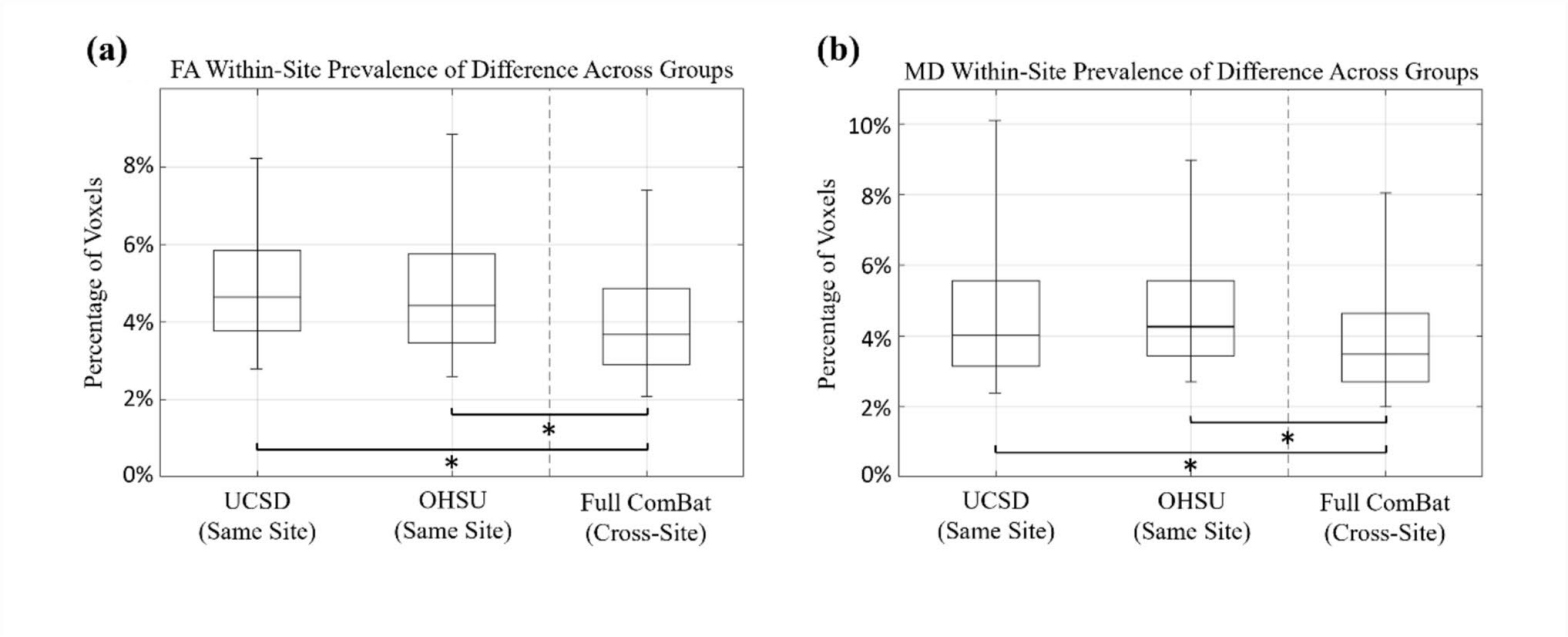
Prevalence of significant voxel-wise **a** FA and **b** MD differences between two randomly sampled groups (*n* = 20 per group) from the same site. In each figure, boxplots left of the dotted line display distributions (based on 5000 repeated trials) of the percentage of WM voxels significantly different across two groups sampled from the same site, while the boxplot right of the dotted line displays that of two groups sampled from each of the two sites after full ComBat was applied to the entire dataset. Asterisks indicate significant differences in median (*p < *0.001; Wilcoxon rank sum test)

Results illustrating the prevalence of significant voxel-wise differences across groups sampled from the same site (for unharmonized data) are displayed in Fig. 7.

## Discussion

In this study, we assessed the harmonization of multisite DTI data using T-ComBat, a method for transferring ComBat harmonization parameters—computed based on a presumed original cohort of ${n}_{\mathrm{Train}}$ subjects per site—to harmonize an added ${n}_{\mathrm{Test}}$ new subjects per site. The utility of this method lies in the ability to harmonize new subject data (from previously observed scanners) without needing to recompute harmonization of previously analyzed subject data. This is beneficial for longitudinal studies in which intermediate analyses may be conducted partway through the collection of data, allowing for continued addition to the harmonized data pool without alteration of previously recorded results. Overall, results indicated that T-ComBat yielded reduced site-based differences in FA and MD, but the harmonizing performance of T-ComBat did not reach the performance of the traditional ComBat algorithm applied to the entire dataset. A limited number of new subject data (in the range of 0-25% more subjects, depending on whether FA or MD was assessed and depending on the size of ${n}_{Train}$) could be added and harmonized via T-ComBat and still yield a sufficiently harmonized total dataset, though exceeding this limit appears to produce a total dataset that is not ideally harmonized.

### T-ComBat Performance Relative to ${{\boldsymbol{n}}}_{\mathbf{t}\mathbf{r}\mathbf{a}\mathbf{i}\mathbf{n}}$ and Full ComBat

The significance of the multiple linear regression association of site with WM FA and MD illustrates the presence of systemic bias in FA and MD values across sites in the unharmonized data, a fact also demonstrated in multiple prior studies [18, 30, 73]. T-ComBat improves the harmonization of data across scanners, and the harmonization performance of T-ComBat improves as the number of training subjects (${n}_{\mathrm{train}}$) is increased. However, application of the full ComBat algorithm to the entire dataset consistently produced fewer voxels (compared to T-ComBat) exhibiting significant differences across sites after harmonization, indicating better performance from full ComBat. This performance difference is consistent with observations from Da-Ano et al. [57], who also observed slightly decreased harmonization performance using the T-ComBat methodology to transfer harmonization parameters to harmonize new multisite radiomics feature data. They noted that the T-ComBat and full ComBat approaches both produced similar performance when used to build predictive models via various machine-learning techniques, suggesting that the decreased T-ComBat harmonization performance may be acceptable in some applications. The current study’s documentation of T-ComBat performance using the overlapping index $\eta$ (which can be interpreted similarly to other normalized measures such as correlation coefficients or *R*^*2*^ [71]) and $PwFC$ provide an estimation of the confidence with which one may expect T-ComBat to perform similarly to full ComBat, though the level of confidence which is acceptable may vary between applications.

The similarity in performance between T-ComBat and full ComBat was greater when assessing ROI-averaged FA and MD than when assessing the voxel-wise values. After observing this result, we hypothesized that T-ComBat may perform better if the ROI-averaged FA or MD values were themselves harmonized (as opposed to harmonizing voxel-wise data). A brief experiment was conducted to test this hypothesis on FA data, with results documented in SM Figure S2. The results indicated an improvement in performance in terms of percentage of ROIs exhibiting differences across sites after T-ComBat (see Figure S2a) but yielded mixed results with respect to the performance of T-ComBat compared with full ComBat conducted on ROI-averaged FA values (higher *PwFC* but lower overlapping index $\eta$; see SM Figure S2c). These results suggest that T-ComBat may be more effective when applied to harmonize the data on a finer level (e.g., voxel-wise FA and MD maps) than the metric of interest (e.g., ROI-averaged FA or MD). The horizontal lines marking the average (over all voxels) difference between sites in Fig. 2 illustrate that, particularly for FA, T-ComBat does reduce voxel-averaged difference between sites over the whole brain (the horizontal mean line moved toward zero for the harmonized plots). Given this global reduction in site-to-site differences, it may be that the ROI-averaged metrics tend to average out remaining voxel-wise differences across sites and produce harmonization closer to that of full ComBat.

It is worth noting that the application of ComBat to the full dataset produced rates of across-site differences that were slightly lower than those detected between groups within the same site. This result suggests that the ComBat algorithm may cause a small degree of over-harmonization, potentially removing data variations that are not solely due to site differences. Further assessment of this phenomenon was outside the scope of this study but should be further explored to ensure the effectiveness of the ComBat algorithm.

### Acceptable DTI Cohort Expansion via T-ComBat

Analysis of T-ComBat’s harmonization performance as assessed on the *combined* training and test group pool for varying ${n}_{\mathrm{Train}}$ and ${n}_{\mathrm{Test}}$ pairs revealed that, as more subject data were included in the training group for both FA and MD data, more new subject data could be added to the pool (harmonized via T-ComBat) and produce full harmonization. FA data harmonized via T-ComBat could consistently accommodate a 10-25% increase in cohort size, while MD data could only accommodate a 0-10% increase. For FA with ${n}_{\mathrm{Train}}$ greater than 70 subjects per site, an approximate trend appeared in which ${n}_{\mathrm{Test}}$ could be increased at about half the rate that ${n}_{\mathrm{Train}}$ was increased while still maintaining full harmonization. If investigators only wish to expand their DTI subject cohort by the proportions described (around 10–25% for FA data, 0–10% for MD data), then T-ComBat may be reasonably expected to produce a full, combined cohort pool of data which does not exhibit significant differences across site. These limits reflect a threshold for adding test subjects to a data pool, where additions beyond these limits represent instances where the pool has obtained enough new information to warrant reharmonization via the traditional ComBat algorithm. We note that Da-ano et al. [57] successfully applied the T-ComBat methodology to a cohort with ${n}_{\mathrm{Train}}=142$ and ${n}_{\mathrm{Test}}=57$ (roughly a 40% cohort increase relative to the initial training group) to harmonize radiomics features across sites, suggesting that the level of acceptable cohort expansion is relative to the data being harmonized.

### Brain Regions Remaining Unharmonized after T-ComBat

Analysis of the frequency with which voxels remained unharmonized at different brain locations suggested that voxels were more likely to remain unharmonized if the voxel fell in an area likely to suffer from partial volume effects (PVE). The frequency of a voxel remaining unharmonized was notably higher in regions neighboring ventricles (particularly toward the posterior end of the lateral ventricles) and toward the edges of WM regions in the cerebellum, each of which is a region where PVE is possible. Further, registration of individual subject brain volumes can result in misalignment of brain tissue regions that hinders voxel-level assessments [74], as the tissue present in a given voxel may vary across subjects’ co-registered volumes. FA and MD measurements are likely to differ between different tissue types [11], so areas in which different tissues are present in a given voxel across subjects are likely to suffer from poor estimates of the local harmonization parameters. Tract-based spatial statistics have been proposed as a method to mitigate registration error by projecting DTI metrics onto a WM skeleton co-registered between subjects [75], a technique applied during a previous study of the NCANDA-A cohort [59]. However, this method suffers from drawbacks related to projection error and an inability to conduct voxel-level assessments [76].

Given the variation in FA and MD values between tissue types, one may hypothesize that running T-ComBat on FA or MD data from only WM voxels (as opposed to whole-brain maps as done in this study) may yield improved harmonization of the WM voxels, which may be sufficient for studies of DTI localized to ROIs within WM. This hypothesis was tested by conducting a brief experiment where the only data entered into T-ComBat were FA data from voxels classified as WM (using FSL *fast*) across all subjects. Details on the results of this experiment are presented in SM Figure S3. The adjustment produced a reduction in the percentage of voxels remaining unharmonized after T-ComBat but resulted in reduced performance relative to full ComBat as measured by the overlapping index $\eta$ and $PwFC$ for most sizes of ${n}_{\mathrm{Train}}$. This suggests that harmonization of exclusively WM voxel data may improve the performance of T-ComBat or ComBat compared to application to whole-brain data, but this adjustment does not resolve the performance gap between T-ComBat and ComBat. Thus, variation in tissue types within tissue-boundary voxels across subjects is likely not the cause of T-ComBat’s lower performance than full ComBat.

In addition, given that statistically significant differences across sites were tested at the *p < *0.05 level, a stronger argument for complete harmonization could be made if, after harmonization, all voxels exhibited statistically significant differences for less than 5% of iterations. However, our study’s results demonstrated that, although the regions likely to experience PVE exhibited the highest incidence of remaining unharmonized, many voxels throughout the remainder of the JHU atlas also remained unharmonized for more than 5% of the tested T-ComBat iterations. Therefore, any incomplete harmonization from T-ComBat is not limited to voxels likely to suffer from PVE.

### Limitations

One limitation of the current study is the sample size of the datasets from the two MRI scan sites assessed. While samples of 180 (UCSD) and 134 (OHSU) represent a moderately large sample size for MRI studies, these sizes still limit the size of the training and test groups that can be assessed when evaluating T-ComBat. To maintain consistent comparisons in performance of T-ComBat across different ${n}_{\mathrm{Train}}$ values, it is necessary to assess the performance of T-ComBat on a consistent test group size (${n}_{\mathrm{Test}}=20$ subjects per site was used for the first analyses conducted in the current study). A higher quantity of subject data would increase the number of subjects that could be included in the test pool, which would increase the confidence level of the observed existence or lack of statistically significant difference in FA or MD values across sites after harmonization. Additionally, the results of the current study—particularly the guidelines for acceptable cohort expansion via T-ComBat—are limited to applications for harmonizing FA and MD values, specifically. Further analysis is needed to assess the feasibility of T-ComBat for harmonization of other imaging metrics. Special consideration should be given to the types of biases that may be present in other types of imaging data or metrics, as this may impact the acceptable minimum number of training subjects used in the T-ComBat approach.

## Conclusion

In this study, T-ComBat was assessed to determine the feasibility of using ComBat harmonization parameters, computed using an existing “training” cohort of subjects from two imaging sites, to harmonize DTI measures of new “test” subjects across sites. The results demonstrated that T-ComBat harmonization performance does not fully reach that of full ComBat applied to an entire dataset of FA or MD measures, though T-ComBat can still be used to harmonize a limited number of new subject data while producing a combined (old and new subjects) dataset that does not exhibit statistically significant differences across sites. Specifically, for harmonization of FA values, adding new subjects representing approximately 25% or less of the original training group size appeared to produce acceptable harmonization of the total expanded dataset if enough subjects (around 80 or more per site) were included in the original training group. For MD values, this expansion was limited to less than around 10% of the original training group size. Voxels that remained unharmonized after application of T-ComBat often appeared in areas likely to suffer from PVE, highlighting the need for careful consideration of registration methods when conducting voxel-level assessments. These results provide guidelines for the acceptable application of T-ComBat in longitudinal studies to harmonize newly added DTI subject data from known imaging sites without the need for reharmonization of previously included subject data.

## Supplementary Information

Below is the link to the electronic supplementary material.

Supplementary Material (PDF 980 KB)
