# Supplementary material for: Evaluating Transferability of ComBat Harmonization of Diffusion Tensor Magnetic Resonance Imaging Data
Source: Ann Biomed Eng. 2025 Nov 7;54(2):479–94. doi: 10.1007/s10439-025-03886-w (PMC12852171; doi:10.1007/s10439-025-03886-w)
Supplement: Supplementary file 1 — Supplementary Material (PDF 980 KB) [file 10439_2025_3886_MOESM1_ESM.pdf]

Supplementary Material - Evaluating Transferability of ComBat Harmonization of Diffusion Tensor Magnetic Resonance Imaging Data

Journal: Annals of Biomedical Engineering

Authors: Bradley Fitzgerald[1,2] & Thomas M. Talavage[2,3]
[1]School of Engineering, Samarkand International University of Technology, Samarkand, Uzbekistan
[2]Elmore Family School of Electrical and Computer Engineering, Purdue University, West Lafayette, IN, USA
[3]Department of Biomedical Engineering, University of Cincinnati, Cincinnati, OH, USA

Corresponding Author: Bradley Fitzgerald (bfitzgerald8812@gmail.com)

Section 1. Supplementary Analysis – Preservation of Dependence Between FA/MD and Covariates

In addition to the evaluation of T-ComBat's harmonizing performance discussed in the main manuscript for this study, it was of interest to assess T-ComBat's ability to preserve interaction between the variable of interest (in this case, FA or MD) and relevant covariates. To this end, for each of the ROI-level iterative assessments described in the main manuscript, tests were run to measure dependence between ROI-averaged FA values (for all test subjects across both sites) and sex (using a two-sample *t*-test across sex) and between ROI-average FA values and age (using Pearson's correlation coefficient; Matlab *corr*), with each recorded as significant when $p < 0.05$. The percentage of ROIs producing statistical significance during each trial was recorded, producing 1000 performance samples for each level of n_{train} as well as 5000 performance samples for the unharmonized data and the full ComBat harmonized data assessments. The distribution of percent ROI samples for each size of n_{train} was compared against that of the unharmonized distribution and the full ComBat harmonized distribution using Wilcoxon signed rank tests with Bonferroni correction for 2 tests per T-ComBat distribution ($p_{Bonferroni} < 0.05$ indicated significant difference).

Supplementary Figure S1 displays results of assessments examining T-Combat's ability to preserve statistical dependence between relevant biological covariates and FA/MD as compared

to full Combat harmonization and no harmonization (tables displaying the specific rates of statistical dependence for each ROI are provided in the Supplementary Material Tables 1-4). For FA, based on the 1000 T-ComBat trials conducted per n_{train} level, the resulting T-ComBat distributions (representing the proportion of ROIs for which averaged FA was significantly associated with sex or age) always exhibited a statistically significant difference with that of the unharmonized data (Wilcoxon signed rank test, $p_{Bonferroni} < 0.05$), indicating increased likelihood of observing a significant relationship after T-ComBat compared to the unharmonized data. FA T-ComBat distributions did not show any statistically significant difference compared with that of full ComBat. For ROI-averaged MD associated with sex, all T-ComBat distributions showed significant differences compared with the unharmonized data and some (but not all) T-ComBat distributions also exhibited statistically significant difference with that of the full ComBat-harmonized data. Only a few T-ComBat distributions exhibited significant differences with the unharmonized data, and none with the full ComBat distribution, when assessing differences across sex.

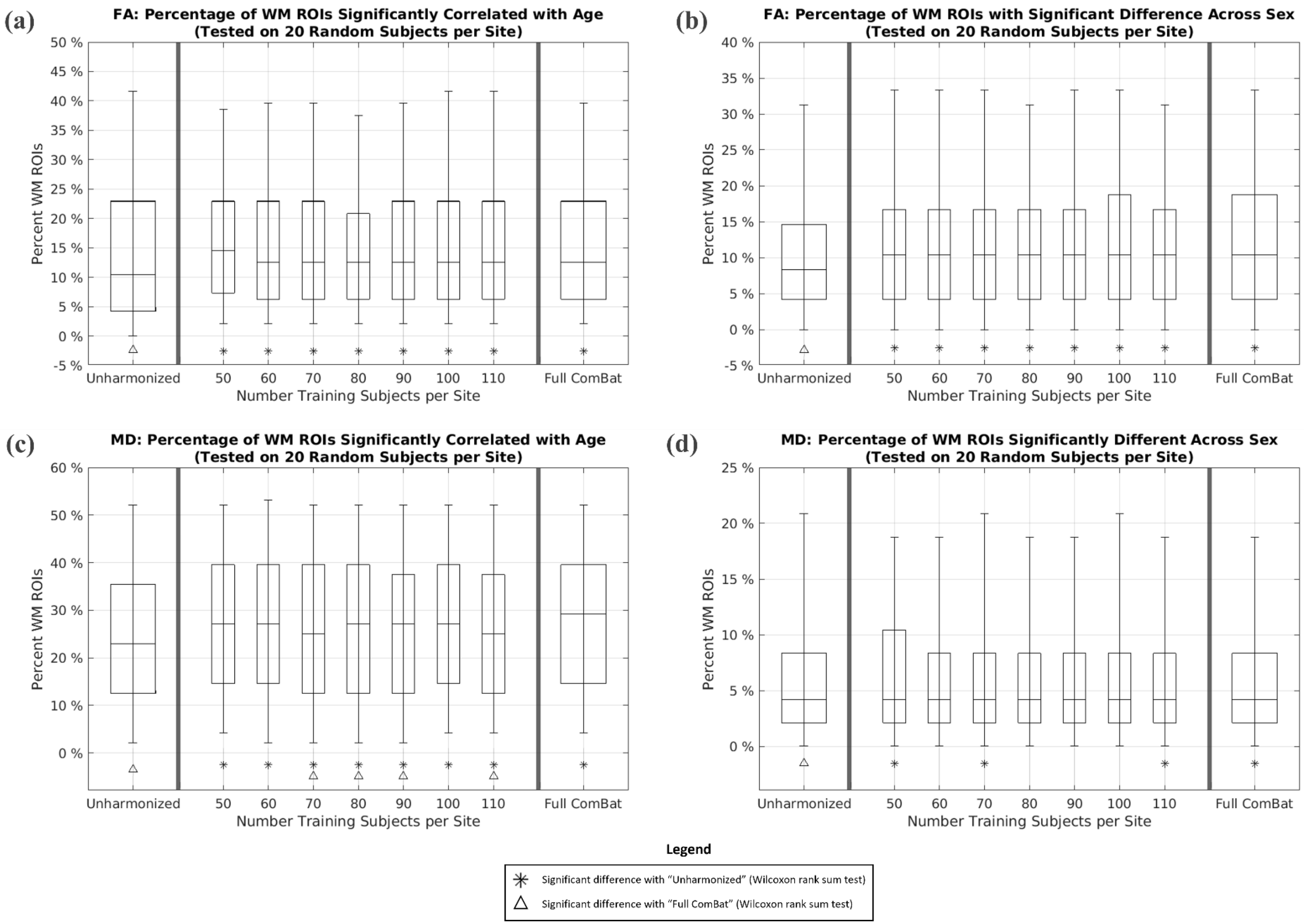
(a)
FA: Percentage of WM ROIs Significantly Correlated with Age
(Tested on 20 Random Subjects per Site)
Percent WM ROIs
Number Training Subjects per Site
Unharmonized
Full ComBat
(b)
FA: Percentage of WM ROIs with Significant Difference Across Sex
(Tested on 20 Random Subjects per Site)
(c)
MD: Percentage of WM ROIs Significantly Correlated with Age
(Tested on 20 Random Subjects per Site)
(d)
MD: Percentage of WM ROIs Significantly Different Across Sex
(Tested on 20 Random Subjects per Site)
Legend
Significant difference with "Unharmonized" (Wilcoxon rank sum test)
Significant difference with "Full ComBat" (Wilcoxon rank sum test)

Figure S1. Frequency of ROI-averaged FA/MD significant association with age and sex. **(a)** Boxplots in the middle plot region represent results of the 1000 T-ComBat trials of FA harmonization (based on same trials as reported in Figure 3c in the main manuscript), reporting the percentage of JHU WM ROIs for which averaged FA was significantly correlated with age (Pearson's correlation, $p < 0.05$) in the test group after T-ComBat harmonization. Boxplots on the left and right side of the plot represent the results of 5000 trials testing for the same FA-age association tested on a same-size test group before harmonization (Unharmonized) and after full harmonization using the full 314-subject dataset (Full ComBat). **(b)** Boxplots reporting similar results to (a), except here assessing significant difference in ROI-averaged FA across sex (using two-sample *t*-tests, $p < 0.05$). **(c)** Boxplots reporting similar results to (a), except here assessing significant correlation between ROI-averaged MD and age. **(d)** Boxplots reporting similar results to (b), except here assessing significant difference in ROI-averaged MD across sex. In all boxplots, lower and upper whiskers mark the 5th and 95th percentiles, respectively.

Table S1. Rates of significant association between FA and age as n_{Train} varies, compared against that of unharmonized FA data and data after full ComBat.

ROI Number	Region	Percentage of Trials Producing Significant Correlation Between ROI-Averaged FA and Age								
		Unharmonized	T-ComBat with n_{Train} Training Subjects per Site							Full ComBat
			50	60	70	80	90	100	110	
1	middle cerebellar peduncle	8.7%	9.5%	7.8%	7.7%	8.9%	7.0%	9.0%	7.3%	8.6%
2	pontine crossing tract	4.3%	3.0%	2.6%	3.4%	4.4%	3.0%	3.5%	3.4%	2.9%
3	genu of corpus callosum	5.9%	7.1%	5.5%	5.1%	5.7%	4.1%	5.4%	5.6%	5.4%
4	body of corpus callosum	6.3%	7.3%	7.5%	8.4%	7.6%	8.5%	7.4%	6.9%	7.0%
5	splenium of corpus callosum	32.5%	40.3%	37.5%	41.9%	40.5%	37.8%	39.3%	36.0%	39.0%
6	fornix	3.4%	3.1%	3.8%	4.9%	4.0%	3.9%	3.4%	3.9%	3.7%
7	corticospinal tract r	5.3%	4.0%	3.9%	4.1%	3.9%	4.3%	4.1%	3.9%	4.0%
8	corticospinal tract l	4.0%	2.7%	2.5%	2.8%	3.5%	2.8%	3.0%	3.0%	3.1%
9	medial lemniscus r	10.9%	9.4%	9.7%	10.4%	9.6%	8.0%	7.8%	10.2%	10.1%
10	medial lemniscus l	12.3%	9.4%	9.7%	11.3%	9.0%	9.8%	9.8%	11.5%	11.0%
11	inferior cerebellar peduncle r	5.4%	4.5%	6.3%	6.0%	4.6%	6.3%	5.7%	5.3%	5.5%
12	inferior cerebellar peduncle l	7.7%	8.7%	7.7%	7.4%	6.2%	6.6%	8.0%	7.0%	8.0%
13	superior cerebellar peduncle r	7.4%	5.5%	5.3%	5.9%	5.8%	6.3%	4.4%	7.0%	6.4%
14	superior cerebellar peduncle l	7.4%	6.0%	6.7%	6.8%	6.5%	6.3%	6.0%	8.0%	6.1%
15	cerebral peduncle r	54.9%	53.9%	56.2%	55.3%	53.2%	55.1%	52.2%	55.6%	56.7%
16	cerebral peduncle l	38.9%	53.0%	55.5%	52.7%	54.0%	53.9%	52.5%	55.4%	54.6%
17	anterior limb of internal capsule r	23.6%	26.3%	25.2%	26.5%	26.1%	29.5%	27.7%	27.3%	26.3%
18	anterior limb of internal capsule l	16.0%	18.9%	18.2%	17.7%	16.4%	18.3%	16.7%	17.7%	17.5%
19	posterior limb of internal capsule r	11.3%	9.9%	9.0%	10.6%	9.8%	11.5%	13.4%	10.1%	10.4%
20	posterior limb of internal capsule l	16.3%	16.4%	16.4%	16.6%	15.1%	17.3%	19.4%	16.2%	16.3%
21	retrolenticular part of internal capsule r	13.1%	12.7%	12.7%	13.7%	12.2%	11.4%	11.0%	13.3%	12.0%
22	retrolenticular part of internal capsule l	11.1%	13.0%	10.4%	12.5%	11.8%	12.4%	12.6%	11.9%	12.8%
23	anterior corona radiata r	11.2%	11.9%	10.7%	10.3%	8.7%	9.7%	11.3%	8.6%	10.3%

24	anterior corona radiata l	17.8%	20.2%	18.6%	17.8%	18.0%	17.4%	16.8%	15.6%	17.7%
25	superior corona radiata r	31.7%	30.8%	31.0%	31.4%	29.5%	31.9%	30.9%	28.5%	31.1%
26	superior corona radiata l	32.2%	34.5%	34.7%	35.6%	35.9%	37.1%	37.1%	33.6%	37.1%
27	posterior corona radiata r	13.4%	11.9%	11.3%	11.2%	11.1%	13.1%	12.8%	11.9%	12.1%
28	posterior corona radiata l	12.3%	10.7%	9.1%	8.4%	10.8%	11.0%	9.9%	10.6%	10.4%
29	posterior thalamic radiation	10.2%	9.9%	11.0%	12.8%	10.3%	11.5%	9.0%	11.5%	10.8%
30	posterior thalamic radiation	15.1%	14.4%	15.8%	17.0%	14.5%	16.3%	16.5%	15.3%	15.5%
31	sagittal stratum	29.3%	35.5%	38.4%	37.3%	35.7%	36.7%	35.6%	36.1%	37.1%
32	sagittal stratum	19.1%	28.7%	32.0%	33.3%	30.9%	31.1%	27.8%	30.2%	29.9%
33	external capsule r	28.2%	29.2%	26.4%	27.0%	26.1%	26.2%	26.2%	28.9%	28.8%
34	external capsule l	42.5%	42.6%	38.9%	39.3%	37.6%	39.3%	38.4%	40.3%	40.8%
35	cingulum	17.7%	19.2%	17.7%	17.6%	14.3%	19.7%	16.6%	18.6%	18.1%
36	cingulum	9.2%	12.5%	12.6%	14.7%	10.7%	13.4%	10.9%	12.2%	13.0%
37	cingulum	5.3%	6.7%	5.9%	5.4%	5.1%	6.1%	4.4%	5.4%	5.6%
38	cingulum	4.6%	4.8%	4.5%	5.2%	4.9%	4.7%	5.8%	3.7%	4.6%
39	fornix	15.5%	22.6%	20.3%	19.5%	19.1%	19.1%	21.0%	21.2%	21.0%
40	fornix	20.8%	28.5%	27.2%	26.6%	24.1%	27.7%	26.5%	26.3%	26.9%
41	superior longitudinal fasciculus r	12.7%	11.4%	10.6%	10.6%	11.9%	12.4%	9.9%	10.8%	11.7%
42	superior longitudinal fasciculus l	13.1%	9.9%	13.1%	11.2%	12.6%	12.7%	10.9%	12.8%	12.9%
43	superior fronto-occipital fasciculus	4.3%	4.0%	4.4%	5.3%	3.5%	4.5%	4.2%	3.2%	4.0%
44	superior fronto-occipital fasciculus	3.9%	3.9%	4.4%	5.7%	2.3%	4.5%	3.6%	4.6%	4.2%
45	uncinate fasciculus r	13.4%	9.9%	10.3%	10.8%	8.9%	10.7%	11.3%	10.5%	12.0%
46	uncinate fasciculus l	12.9%	10.0%	11.2%	10.5%	10.1%	10.9%	10.2%	11.7%	11.6%
47	tapetum r	11.6%	17.0%	16.4%	17.3%	17.2%	16.5%	18.2%	16.9%	16.7%
48	tapetum l	14.2%	12.5%	13.9%	12.1%	13.9%	12.6%	13.0%	12.1%	12.6%

Table S2. Rates of significant association between FA and sex as n_{Train} varies, compared against that of unharmonized FA data and data after full ComBat.

ROI Number	Region	Percentage of Trials Producing Significant Difference in ROI-Averaged FA Across Sex								
		Unharmonized	T-ComBat with n_{Train} Training Subjects per Site							Full ComBat
			50	60	70	80	90	100	110	
1	middle cerebellar peduncle	10.1%	9.8%	9.0%	10.9%	9.5%	8.5%	9.2%	8.9%	10.4%
2	pontine crossing tract	4.1%	4.5%	4.8%	5.1%	4.9%	4.4%	5.1%	4.7%	4.9%
3	genu of corpus callosum	6.8%	9.9%	7.6%	9.4%	9.3%	8.4%	9.4%	9.0%	9.1%
4	body of corpus callosum	18.5%	17.3%	17.8%	19.0%	17.3%	16.7%	18.2%	16.9%	19.0%
5	splenium of corpus callosum	3.7%	3.6%	4.3%	4.6%	3.5%	3.2%	4.1%	2.6%	3.6%
6	fornix	3.8%	3.4%	3.9%	4.6%	5.0%	3.8%	4.7%	4.6%	3.8%
7	corticospinal tract r	3.5%	3.6%	4.0%	3.4%	4.2%	5.1%	3.7%	2.8%	3.6%
8	corticospinal tract l	3.5%	2.8%	3.6%	3.6%	3.5%	3.6%	3.5%	2.5%	3.8%
9	medial lemniscus r	11.7%	15.0%	17.0%	15.3%	15.1%	19.6%	18.6%	15.2%	17.0%
10	medial lemniscus l	9.1%	10.9%	12.6%	10.9%	11.6%	14.3%	14.2%	11.6%	13.0%
11	inferior cerebellar peduncle r	10.9%	9.9%	10.1%	11.5%	12.6%	10.7%	10.6%	9.7%	11.0%
12	inferior cerebellar peduncle l	13.2%	11.1%	14.0%	12.8%	12.7%	11.8%	12.5%	12.0%	12.6%
13	superior cerebellar peduncle r	8.8%	15.7%	15.3%	15.1%	17.1%	16.7%	14.7%	16.3%	14.8%
14	superior cerebellar peduncle l	10.0%	16.7%	15.9%	14.5%	15.6%	15.8%	15.4%	15.8%	15.4%
15	cerebral peduncle r	15.0%	18.8%	17.2%	16.7%	17.9%	18.4%	18.0%	17.7%	18.0%
16	cerebral peduncle l	7.6%	13.4%	11.7%	10.5%	11.6%	11.9%	13.3%	12.2%	12.4%
17	anterior limb of internal capsule r	52.7%	53.5%	52.4%	51.6%	52.5%	51.5%	52.4%	54.0%	55.3%
18	anterior limb of internal capsule l	39.6%	39.6%	38.0%	38.1%	39.0%	38.3%	39.2%	39.7%	42.0%
19	posterior limb of internal capsule r	15.4%	16.8%	18.6%	17.6%	19.7%	19.8%	17.1%	17.0%	16.9%
20	posterior limb of internal capsule l	10.1%	14.7%	16.3%	14.9%	15.6%	15.1%	15.3%	15.3%	13.6%
21	retrolenticular part of internal capsule r	6.6%	8.6%	7.5%	7.3%	8.1%	7.5%	8.2%	8.5%	8.5%
22	retrolenticular part of internal capsule l	6.5%	10.6%	9.7%	10.3%	11.6%	10.5%	12.0%	10.1%	10.7%
23	anterior corona radiata r	5.6%	5.8%	5.5%	5.7%	5.1%	6.6%	5.7%	5.2%	6.4%

24	anterior corona radiata l	6.8%	6.1%	7.8%	6.2%	5.4%	7.1%	7.3%	7.3%	7.9%
25	superior corona radiata r	4.1%	3.0%	3.6%	3.3%	4.9%	3.6%	3.7%	2.4%	3.5%
26	superior corona radiata l	5.6%	8.3%	6.4%	6.9%	8.2%	7.8%	8.0%	6.0%	8.4%
27	posterior corona radiata r	4.6%	4.1%	5.0%	5.5%	5.2%	4.7%	6.2%	3.8%	5.0%
28	posterior corona radiata l	4.9%	5.6%	5.4%	5.7%	6.7%	5.7%	5.8%	5.0%	6.0%
29	posterior thalamic radiation	3.9%	5.2%	3.7%	3.6%	4.1%	4.7%	5.7%	4.6%	4.7%
30	posterior thalamic radiation	4.7%	6.6%	4.6%	5.2%	6.1%	6.3%	6.4%	4.9%	6.2%
31	sagittal stratum	5.3%	6.5%	5.5%	6.7%	6.5%	6.7%	5.9%	6.2%	6.8%
32	sagittal stratum	5.6%	8.9%	6.7%	7.8%	7.5%	7.9%	8.7%	7.6%	8.7%
33	external capsule r	10.1%	10.4%	9.1%	10.2%	9.5%	9.0%	8.9%	10.8%	9.2%
34	external capsule l	8.7%	9.8%	8.0%	8.5%	8.6%	9.7%	11.3%	9.0%	9.9%
35	cingulum	24.3%	25.7%	23.1%	24.4%	24.1%	24.2%	23.9%	24.7%	24.8%
36	cingulum	24.9%	27.9%	27.7%	25.5%	27.3%	26.3%	26.3%	27.6%	27.7%
37	cingulum	17.5%	15.8%	18.4%	15.5%	15.4%	16.4%	16.9%	17.0%	17.0%
38	cingulum	11.1%	11.3%	12.2%	9.6%	9.1%	11.3%	10.7%	9.9%	10.7%
39	fornix	4.1%	5.4%	6.2%	4.2%	4.6%	4.1%	5.7%	4.4%	5.2%
40	fornix	5.1%	7.4%	8.4%	7.2%	7.3%	7.9%	9.7%	8.6%	8.4%
41	superior longitudinal fasciculus r	16.8%	22.2%	17.6%	21.8%	20.9%	21.4%	20.7%	21.0%	21.8%
42	superior longitudinal fasciculus l	7.7%	12.7%	11.7%	11.5%	10.4%	12.6%	11.5%	12.5%	12.6%
43	superior fronto-occipital fasciculus	18.1%	17.8%	19.0%	19.7%	20.0%	20.2%	19.0%	20.1%	18.9%
44	superior fronto-occipital fasciculus	27.1%	25.5%	25.6%	27.7%	26.9%	25.0%	23.7%	28.2%	27.8%
45	uncinate fasciculus r	15.0%	15.0%	14.1%	14.2%	13.2%	13.3%	14.6%	14.6%	14.3%
46	uncinate fasciculus l	11.5%	11.4%	11.2%	10.4%	9.9%	9.6%	11.9%	9.7%	10.9%
47	tapetum r	4.1%	4.5%	4.6%	3.7%	3.8%	4.3%	4.8%	4.8%	4.5%
48	tapetum l	4.2%	4.8%	3.5%	4.4%	5.0%	4.2%	3.9%	4.1%	3.9%

Table S3. Rates of significant association between MD and age as n_{Train} varies, compared against that of unharmonized FA data and data after full ComBat.

ROI Number	Region	Percentage of Trials Producing Significant Correlation Between ROI-Averaged MD and Age								
		Unharmonized	T-ComBat with n_{Train} Training Subjects per Site							Full ComBat
			50	60	70	80	90	100	110	
1	middle cerebellar peduncle	7.4%	6.9%	6.8%	6.8%	6.3%	7.0%	5.8%	4.7%	6.4%
2	pontine crossing tract	9.6%	12.8%	11.4%	11.1%	9.8%	11.9%	11.7%	10.0%	12.3%
3	genu of corpus callosum	4.9%	4.3%	4.5%	4.7%	5.2%	5.0%	6.1%	6.3%	5.4%
4	body of corpus callosum	24.6%	35.2%	38.2%	36.1%	36.5%	35.1%	35.3%	38.3%	37.5%
5	splenium of corpus callosum	13.0%	12.6%	11.9%	13.4%	10.9%	10.9%	12.3%	10.7%	12.7%
6	fornix	5.8%	6.0%	5.5%	4.9%	4.6%	4.7%	6.3%	5.7%	5.5%
7	corticospinal tract r	14.0%	14.9%	14.3%	12.2%	12.7%	12.1%	13.2%	12.1%	14.1%
8	corticospinal tract l	13.8%	14.3%	14.0%	11.9%	12.8%	11.9%	12.7%	10.6%	13.8%
9	medial lemniscus r	9.8%	9.2%	9.1%	9.1%	9.2%	8.6%	8.4%	9.3%	8.8%
10	medial lemniscus l	10.9%	9.7%	10.4%	9.3%	9.9%	8.6%	9.6%	8.6%	9.5%
11	inferior cerebellar peduncle r	4.5%	6.2%	4.1%	5.6%	4.4%	5.3%	5.0%	5.2%	5.7%
12	inferior cerebellar peduncle l	3.4%	4.5%	3.4%	2.8%	3.2%	3.1%	2.9%	3.0%	3.9%
13	superior cerebellar peduncle r	4.5%	3.7%	3.3%	2.3%	3.2%	3.3%	2.8%	3.0%	3.5%
14	superior cerebellar peduncle l	4.8%	4.5%	3.4%	3.1%	4.2%	3.8%	4.4%	4.3%	3.8%
15	cerebral peduncle r	22.4%	18.8%	20.1%	20.3%	20.2%	18.7%	21.5%	19.5%	20.4%
16	cerebral peduncle l	15.3%	19.9%	23.4%	19.0%	20.2%	20.1%	21.7%	18.8%	20.0%
17	anterior limb of internal capsule r	62.1%	61.3%	62.2%	60.2%	61.3%	61.1%	63.7%	59.6%	64.0%
18	anterior limb of internal capsule l	59.1%	60.3%	60.0%	57.7%	55.7%	57.2%	60.2%	58.5%	63.0%
19	posterior limb of internal capsule r	71.5%	69.8%	68.8%	68.2%	67.5%	68.1%	72.2%	69.9%	72.3%
20	posterior limb of internal capsule l	39.0%	60.8%	60.6%	59.3%	59.6%	58.6%	63.3%	61.2%	64.3%
21	retrolenticular part of internal capsule r	57.7%	58.0%	57.4%	55.6%	56.1%	58.4%	60.2%	57.3%	60.5%
22	retrolenticular part of internal capsule l	23.6%	35.9%	36.0%	35.8%	34.6%	35.1%	37.3%	33.9%	37.9%
23	anterior corona radiata r	50.2%	49.9%	49.0%	47.3%	48.3%	48.0%	49.7%	47.9%	52.2%

24	anterior corona radiata l	66.9%	66.2%	65.7%	63.9%	63.9%	63.3%	65.2%	64.3%	67.6%
25	superior corona radiata r	54.0%	59.4%	59.5%	56.4%	58.3%	57.8%	60.7%	56.1%	60.6%
26	superior corona radiata l	31.9%	51.4%	53.4%	49.6%	52.6%	51.0%	54.6%	51.1%	55.7%
27	posterior corona radiata r	48.6%	47.7%	48.1%	45.8%	44.9%	44.7%	49.2%	46.0%	48.7%
28	posterior corona radiata l	33.5%	35.3%	38.0%	32.9%	34.0%	35.6%	36.6%	34.8%	38.1%
29	posterior thalamic radiation	8.3%	9.2%	9.0%	9.0%	10.1%	9.1%	8.2%	8.2%	9.3%
30	posterior thalamic radiation	5.4%	5.3%	5.1%	6.0%	6.5%	5.1%	6.5%	4.6%	5.6%
31	sagittal stratum	21.6%	24.3%	25.0%	23.6%	24.9%	24.6%	25.7%	23.8%	24.2%
32	sagittal stratum	7.0%	9.2%	9.2%	7.6%	8.1%	8.8%	10.6%	11.0%	9.0%
33	external capsule r	40.0%	41.7%	41.5%	43.0%	39.2%	39.8%	42.0%	40.3%	43.2%
34	external capsule l	37.8%	37.7%	39.4%	39.5%	36.9%	34.7%	38.1%	37.3%	39.5%
35	cingulum	51.1%	48.7%	48.8%	49.6%	48.3%	47.5%	49.8%	48.5%	50.1%
36	cingulum	43.0%	47.2%	44.3%	45.3%	44.1%	45.6%	45.3%	44.4%	45.5%
37	cingulum	8.0%	5.1%	6.5%	5.3%	4.2%	6.4%	5.2%	5.7%	5.0%
38	cingulum	12.6%	6.6%	9.9%	10.4%	8.9%	11.1%	8.3%	10.5%	8.8%
39	fornix	12.3%	14.4%	15.8%	13.6%	14.5%	16.6%	14.7%	14.7%	14.5%
40	fornix	13.6%	24.7%	23.7%	23.5%	23.3%	24.4%	25.4%	23.0%	23.8%
41	superior longitudinal fasciculus r	69.0%	69.3%	69.7%	69.1%	66.5%	67.6%	70.4%	67.2%	71.2%
42	superior longitudinal fasciculus l	27.4%	46.4%	49.7%	49.0%	47.3%	47.6%	50.0%	47.8%	52.1%
43	superior fronto-occipital fasciculus	23.5%	22.0%	23.6%	22.8%	21.7%	21.5%	22.5%	21.2%	23.3%
44	superior fronto-occipital fasciculus	17.3%	17.2%	16.4%	18.5%	15.7%	16.0%	20.2%	16.6%	19.4%
45	uncinate fasciculus r	6.6%	9.9%	11.5%	10.1%	8.9%	10.2%	10.3%	9.6%	9.8%
46	uncinate fasciculus l	9.2%	17.3%	17.6%	17.3%	16.1%	16.4%	18.8%	17.8%	18.0%
47	tapetum r	5.5%	4.9%	4.6%	5.5%	5.7%	5.0%	5.8%	5.0%	5.0%
48	tapetum l	6.3%	4.4%	4.0%	3.4%	4.0%	3.4%	5.0%	3.6%	3.7%

Table S4. Rates of significant association between MD and sex as n_{Train} varies, compared against that of unharmonized FA data and data after full ComBat.

ROI Number	Region	Percentage of Trials Producing Significant Difference in ROI-Averaged MD Across Sex								
		Unharmonized	T-ComBat with n_{Train} Training Subjects per Site							Full ComBat
			50	60	70	80	90	100	110	
1	middle cerebellar peduncle	5.2%	4.4%	4.8%	6.8%	4.4%	5.4%	4.9%	4.3%	5.0%
2	pontine crossing tract	10.1%	10.1%	7.8%	10.4%	8.5%	8.1%	8.0%	8.5%	8.4%
3	genu of corpus callosum	4.0%	5.0%	3.3%	3.7%	2.5%	4.0%	2.6%	3.9%	3.6%
4	body of corpus callosum	4.8%	6.8%	4.4%	4.8%	6.2%	5.7%	4.4%	5.2%	5.3%
5	splenium of corpus callosum	4.0%	3.8%	4.4%	3.1%	5.2%	3.0%	4.3%	4.9%	3.7%
6	fornix	3.8%	2.8%	4.0%	3.2%	4.4%	3.8%	3.8%	3.8%	3.7%
7	corticospinal tract r	3.1%	4.8%	3.1%	4.6%	3.9%	4.6%	3.5%	3.9%	3.3%
8	corticospinal tract l	3.1%	4.7%	2.9%	3.9%	4.1%	3.5%	2.4%	3.7%	3.0%
9	medial lemniscus r	6.4%	7.5%	9.1%	7.8%	8.8%	7.8%	7.6%	8.5%	7.7%
10	medial lemniscus l	6.1%	6.2%	8.4%	6.9%	7.9%	7.0%	7.4%	8.1%	7.2%
11	inferior cerebellar peduncle r	4.4%	5.0%	5.1%	5.2%	5.5%	4.5%	4.6%	5.4%	4.4%
12	inferior cerebellar peduncle l	11.9%	12.0%	11.4%	13.5%	12.2%	9.6%	10.6%	13.2%	10.6%
13	superior cerebellar peduncle r	4.6%	4.1%	5.0%	5.0%	4.4%	3.8%	4.9%	5.6%	4.5%
14	superior cerebellar peduncle l	5.1%	5.8%	4.8%	4.9%	4.2%	4.4%	4.8%	5.3%	4.8%
15	cerebral peduncle r	4.4%	6.2%	4.1%	4.1%	4.7%	4.6%	3.3%	5.2%	4.3%
16	cerebral peduncle l	3.6%	4.5%	4.2%	3.1%	3.7%	4.0%	4.2%	4.0%	4.3%
17	anterior limb of internal capsule r	8.3%	7.9%	8.8%	10.5%	9.9%	8.8%	10.4%	8.3%	9.5%
18	anterior limb of internal capsule l	4.4%	4.8%	4.5%	5.3%	5.8%	3.7%	4.6%	5.9%	5.0%
19	posterior limb of internal capsule r	6.3%	6.9%	6.8%	8.0%	4.3%	6.6%	7.1%	6.1%	6.3%
20	posterior limb of internal capsule l	4.8%	5.6%	3.8%	5.7%	4.8%	5.1%	5.7%	5.6%	5.9%
21	retrolenticular part of internal capsule r	4.9%	5.2%	6.1%	6.0%	4.9%	4.7%	6.9%	5.1%	4.9%
22	retrolenticular part of internal capsule l	3.8%	4.2%	4.1%	4.6%	2.8%	3.3%	3.3%	4.3%	4.0%
23	anterior corona radiata r	4.5%	4.0%	4.4%	4.8%	4.6%	3.4%	4.2%	5.4%	4.6%

24	anterior corona radiata l	3.9%	3.3%	3.5%	3.9%	3.5%	2.9%	3.3%	3.4%	3.8%
25	superior corona radiata r	4.6%	3.5%	5.0%	6.3%	4.0%	3.8%	5.2%	4.5%	5.0%
26	superior corona radiata l	4.1%	3.5%	4.2%	5.0%	4.0%	3.7%	3.6%	4.5%	4.6%
27	posterior corona radiata r	3.6%	2.8%	3.5%	3.9%	3.2%	3.0%	4.0%	3.2%	3.7%
28	posterior corona radiata l	6.3%	6.2%	5.9%	5.5%	5.2%	4.9%	5.2%	5.5%	6.4%
29	posterior thalamic radiation	7.9%	9.8%	9.5%	8.4%	10.0%	7.5%	8.7%	8.4%	9.3%
30	posterior thalamic radiation	9.3%	9.0%	7.7%	8.5%	7.0%	7.0%	8.3%	8.3%	8.9%
31	sagittal stratum	3.4%	2.3%	4.1%	4.1%	3.5%	3.2%	3.3%	3.6%	2.9%
32	sagittal stratum	3.7%	3.4%	3.2%	3.5%	3.3%	4.0%	3.2%	4.3%	3.3%
33	external capsule r	4.5%	4.5%	3.4%	5.7%	3.8%	3.3%	4.2%	6.0%	4.2%
34	external capsule l	5.7%	5.1%	4.8%	4.8%	5.6%	4.7%	5.7%	5.8%	5.8%
35	cingulum	29.5%	28.5%	27.3%	29.3%	28.6%	25.1%	29.6%	27.4%	27.5%
36	cingulum	18.6%	20.9%	20.9%	21.3%	21.4%	18.4%	21.4%	19.0%	19.7%
37	cingulum	7.2%	9.2%	7.3%	9.0%	7.1%	7.2%	8.9%	7.9%	7.6%
38	cingulum	5.1%	4.7%	4.4%	5.3%	4.9%	4.6%	3.9%	5.2%	4.3%
39	fornix	4.4%	5.3%	3.3%	3.8%	4.8%	6.3%	4.4%	5.3%	4.7%
40	fornix	5.8%	10.4%	7.3%	8.1%	8.7%	8.2%	7.1%	8.2%	7.9%
41	superior longitudinal fasciculus r	4.1%	3.2%	3.8%	4.9%	3.0%	3.4%	4.3%	3.8%	4.1%
42	superior longitudinal fasciculus l	4.0%	4.2%	4.5%	4.3%	2.6%	3.3%	4.0%	4.5%	3.8%
43	superior fronto-occipital fasciculus	4.2%	4.1%	4.2%	4.5%	4.6%	4.4%	6.1%	4.4%	4.7%
44	superior fronto-occipital fasciculus	4.3%	4.6%	3.0%	4.6%	4.2%	3.8%	3.7%	5.2%	4.4%
45	uncinate fasciculus r	3.8%	3.5%	4.3%	3.7%	3.8%	3.1%	4.4%	4.1%	3.3%
46	uncinate fasciculus l	17.1%	11.7%	11.3%	12.1%	12.6%	10.2%	10.6%	10.7%	9.2%
47	tapetum r	5.3%	7.7%	9.2%	8.0%	8.8%	7.2%	8.5%	9.1%	7.9%
48	tapetum l	5.0%	7.3%	6.8%	5.2%	6.0%	5.5%	5.6%	5.4%	5.3%

Section 2. Additional Supplementary Figures

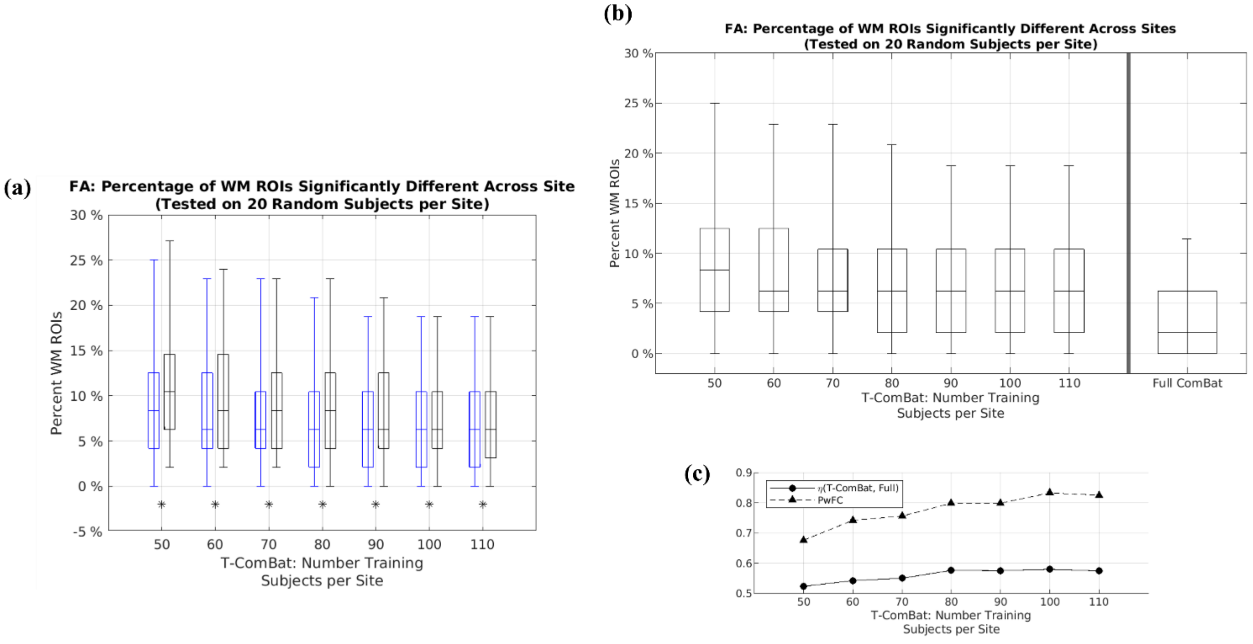

Figure S2. Harmonizing performance of T-ComBat applied to harmonize ROI-averaged FA values. **(a)** Comparison of harmonizing performance of T-ComBat applied only to ROI-averaged FA values (blue; same distributions exhibited in panel b) versus T-ComBat applied on whole-brain voxel-wise FA maps (black; same distributions exhibited in Figure 3c of the main manuscript). T-ComBat applied only to ROI-averaged FA values was assessed by performing 1000 repeated trials per n_{train} value, where n_{train} subjects per site were randomly selected for inclusion in the training group and $n_{test} = 20$ subjects per site were randomly selected for inclusion in the test group. Asterisks indicate statistically significant difference in median between the ROI-averaged T-ComBat performance and the whole-brain voxel-wise T-ComBat performance (Wilcoxon rank sum test, $p < 0.05$). **(b)** T-ComBat, applied to harmonize ROI-averaged FA values, harmonizing performance compared against harmonizing performance of full ComBat. The full ComBat performance distribution is based on 200 trials of randomly selecting 20 subjects per site to assess ROI-averaged FA differences across sites after the ROI-averaged FA data of the entire 314-subject dataset was passed through ComBat. **(c)** Similarity metrics, including overlapping index η and *percent within Full ComBat (PwFC;* i.e., percent of T-ComBat performance measures falling below 95th percentile of full ComBat performance), comparing similarity of T-ComBat and full ComBat harmonization performance.

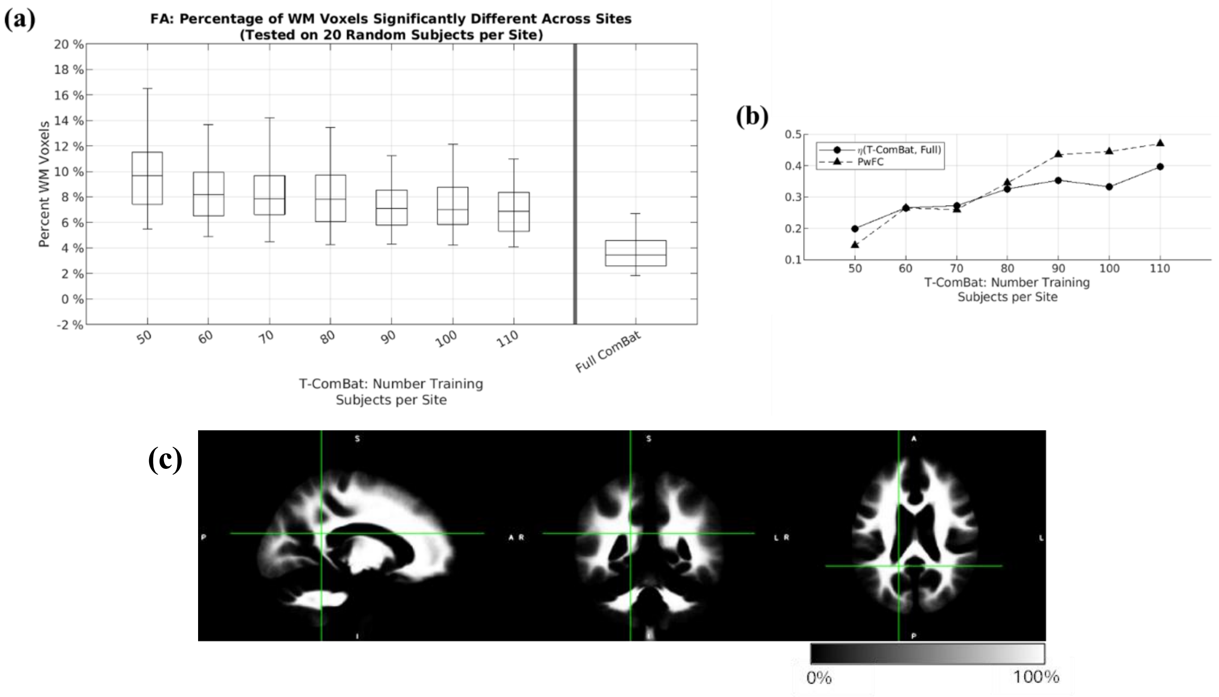

Figure S3. Harmonizing performance of T-ComBat applied to harmonize voxel-wise FA values within exclusive WM mask representing all voxels classified as WM across all subjects. **(a)** T-ComBat, applied to harmonize voxel-wise FA values within the exclusive WM mask (i.e., voxels marked as appearing in 100% of subjects in panel c), harmonizing performance compared against harmonizing performance of full ComBat. T-ComBat applied only to voxel-wise FA values within the exclusive WM mask was assessed by performing 200 repeated trials per n_{train} value, where n_{train} subjects per site were randomly selected for inclusion in the training group and $n_{test} = 20$ subjects per site were randomly selected for inclusion in the test group. The full ComBat performance distribution is based on 1000 trials of randomly selecting 20 subjects per site to assess voxel-wise FA differences across sites after the exclusive WM FA data of the entire 314-subject dataset was passed through ComBat. **(b)** Similarity metrics, including overlapping index η and *percent within Full ComBat (PwFC;* i.e., percent of T-ComBat performance measures falling below 95th percentile of full ComBat performance), comparing similarity of T-ComBat and full ComBat harmonization performance. **(c)** White matter binary masks (computed using FSL *fast*) of all 314 participants averaged after nonlinear registration to MNI space.
